# A Dual-Crosslinked Metallo-elastomer Platform for Architecture-Directed Vascular Remodeling

**DOI:** 10.1007/s42765-026-00744-9

**Published:** 2026-07-04

**Authors:** Narangerel Gantumur, Shuhao Jiao, Xiaochu Ding, Simon Van Herck, Isabella Frangiosa, Emily Kopchick, Caitlin Maureen Purdy, Ty Walker, Aarati Kharal, Ying Grace Chen

**Affiliations:** 1 Department of Biomedical Engineering, Henry M. Rowan College of Engineering, Rowan University, 201 Mullica Hill Road, Glassboro, NJ 08028 USA; 2 Rowan-Virtua School of Translational Biomedical Engineering & Sciences, 42 East Laurel Road, Stratford, NJ 08084 USA; 3 Health Research Institute, Michigan Technological University, H-STEM 238, 1400 Townsend Drive, Houghton, MI 49931 USA; 4 Department of Chemistry, Michigan Technological University, 1400 Townsend Drive, Houghton, MI 49931 USA; 5 Department of Instructive Biomaterials Engineering, MERLN Institute for Technology-Inspired Regenerative Medicine, Maastricht University, Universiteitssingel 40, 6229 ER Maastricht, The Netherlands; 6 Center for Research and Education in Advanced Transportation Engineering Systems (CREATES) of the Henry M. Rowan College of Engineering, Rowan University, 201 Mullica Hill Road, Glassboro, NJ 08028 USA

**Keywords:** Dual-crosslinked metallo-elastomers, Electrowriting, Circumferentially-biased helically wound fiber alignment, Resorbable vascular graft, In situ vascular remodeling

## Abstract

The engineering of durable small-diameter vascular grafts remains constrained by the challenge of simultaneously achieving mechanical robustness, controlled degradation, and instructive scaffold architecture. Here, we report a programmable dual-crosslinked metallo-elastomer platform, poly(1,3-propylene itaconate-co-2,2′-bipyridine-5,5′-dicarboxylate-co-succinate-co-sebacate) (M-PBIS), that integrates dynamic metal–ligand coordination with covalent crosslinking to enable orthogonal control over network mechanics, time-dependent viscoelastic behavior, and processability. PBIS polymers were synthesized by modular step-growth polyesterification, allowing independent tuning of backbone composition, bipyridine ligand density for metal coordination, and alkene (C = C) content for covalent crosslinking. This multidimensional design space allowed systematic tuning of tensile elastic modulus (0.06–3.2 MPa), extensibility (53%–491%), toughness (66–1339 kJ m^−3^), creep resistance, and self-healing behavior, while maintaining controlled hydrolytic degradation and low physiological swelling (< 7%). Rheological analysis established a processing window supportive of conventional melt- and flow-based fabrication methods. To elucidate the role of scaffold architecture in vascular remodeling, M-PBIS was fabricated into small-diameter grafts using either poly(methyl methacrylate) (PMMA)-templated porous structures or electrowritten circumferentially-biased, helically wound fibers. In a rat carotid artery interposition model, electrowritten Zn-PBIS grafts maintained patency and dimensional stability through 21 weeks, supporting organized endothelialization, circumferential smooth muscle alignment, and structured extracellular matrix deposition. In contrast, PMMA-templated porous grafts underwent progressive dilation and structural instability during remodeling. These results demonstrate that dual-crosslinked metallo-elastomers combined with biomimetic circumferentially-biased fiber architecture enable mechanically resilient, biologically adaptive vascular grafts and establish M-PBIS as a manufacturable platform for resorbable small-diameter arterial reconstruction and other load-bearing soft-tissue applications.

**Graphical abstract:**

**Figure:**
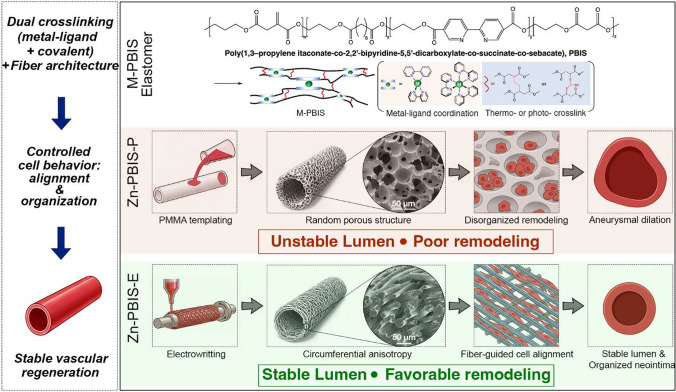

**Supplementary Information:**

The online version contains supplementary material available at 10.1007/s42765-026-00744-9.

## Introduction

Cardiovascular diseases remain the leading cause of morbidity and mortality worldwide, sustaining a major clinical need for vascular bypass and reconstructive procedures [1]. Autologous conduits, such as the saphenous vein and radial artery, remain the clinical gold standard, yet their use is limited by donor-site morbidity, limited availability, and poor vessel quality in patients with diabetes, calcification, or advanced vascular disease [2]. Synthetic grafts based on expanded polytetrafluoroethylene (ePTFE) or polyethylene terephthalate (Dacron) perform reliably in large-diameter vessels but consistently fail in small-diameter arteries (< 6 mm), where thrombosis, compliance mismatch, and poor tissue integration lead to low long-term patency [3]. Despite decades of development, no clinically available synthetic graft has achieved reliable performance for small-diameter arterial reconstruction [4].

Biodegradable, resorbable vascular grafts designed for in situ arterial regeneration have emerged as a promising alternative [5]. In this strategy, acellular scaffolds provide temporary mechanical support while enabling host-cell infiltration, extracellular matrix (ECM) deposition, and progressive remodeling into a functional neovessel, offering practical advantages including off-the-shelf availability, manufacturing reproducibility, and avoidance of cell harvesting [6]. However, durable performance requires synchronizing scaffold resorption with tissue formation, while maintaining lumen geometry under pulsatile arterial loading. If degradation is too rapid, mechanical integrity is lost and dilation or rupture can occur; if too slow, scaffold persistence can impair remodeling and prolong inflammation. Many biodegradable polymers have been explored for vascular grafts, such as poly(lactic acid)/poly(lactic-co-glycolic acid) (PLA/PLGA), polyglycolic acid (PGA), poly(*ε*-caprolactone) (PCL), polydioxanone, and degradable polyurethanes. These polymers face one or more translational limitations, including non-ideal degradation kinetics, acidic degradation byproducts, insufficient elasticity or time-dependent mechanical stability, and inconsistent endothelialization and tissue organization [7]. Elastomeric systems, including poly(glycerol sebacate) (PGS) and its derivatives, represent an important advance by introducing soft, extensible polyesters with improved compliance. However, PGS-based grafts have shown rapid degradation and/or mechanical instability in large-animal studies, leading to dilation and failure [8, 9]. Composite designs that add slowly degrading sheaths can improve durability, but often at the cost of reduced infiltration, impaired integration, and chronic inflammation [9, 10]. Collectively, these results underscore a persistent gap: a resorbable elastomer platform that is simultaneously mechanically robust, degradation-tunable, and manufacturable into architectures that actively guide constructive arterial remodeling.

Dynamic polymer networks based on reversible crosslinks offer an attractive route to meet these competing requirements. In our previous work, we introduced a class of metallo-elastomers, poly(propanediol-co-(hydroxyphenyl methylene)amino-propanediol sebacate) (M-PAS), which leveraged metal–ligand coordination to achieve tunable mechanics and biodegradability [11, 12]. M-PAS grafts demonstrated promising long-term patency and remodeling in rat carotid models, including endothelialization and smooth muscle organization [12]. However, M-PAS lacks melt/solution processability compatible with scalable fabrication methods, such as electrospinning, electrowriting, or 3D printing, necessitating PMMA bead templating for graft manufacture [28]. While PMMA bead templating was suitable for proof-of-concept studies, scale-up to larger grafts revealed structural deficiencies and inconsistent long-term mechanical stability, contributing to pronounced dilation. These challenges motivate the development of a next-generation metallo-elastomer platform that preserves coordination-enabled adaptability while enabling scalable processing and precise architectural control for long-term dimensional stability.

Here, we report a dual-crosslinked metallo-elastomer platform, poly(1,3-propylene itaconate-co-2,2′-bipyridine-5,5′-dicarboxylate-co-succinate-co-sebacate) (M-PBIS), that integrates dynamic metal–ligand coordination with covalent crosslinking to enable orthogonal control over mechanics, degradation, and processability. PBIS is synthesized via modular step-growth polyesterification, incorporating (i) bipyridine ligands for programmable metal coordination, (ii) itaconate units for covalent crosslinking, and (iii) tunable succinate/sebacate content to regulate chain organization and flexibility. We systematically tune network behavior through ligand density, itaconate content, and metal ion identity, and evaluate physicochemical stability, swelling, time-dependent mechanics, and cytocompatibility. Finally, we translate M-PBIS into small-diameter vascular grafts using two distinct architectures, PMMA-templated porous walls and electrowritten circumferentially-biased helically wound fibrous walls, to directly test how scaffold microarchitecture governs remodeling outcomes under arterial hemodynamics. In a rat carotid artery interposition model, electrowritten M-PBIS grafts maintain lumen stability over extended implantation and support organized, circumferentially aligned vascular remodeling, establishing M-PBIS as a manufacturable and programmable platform for resorbable small-diameter vascular graft engineering.

## Results and Discussion

### Design, Synthesis, and Characterization of PBIS Elastomers

#### PBIS Polymer Synthesis and Compositional Control

**Fig. 1:**
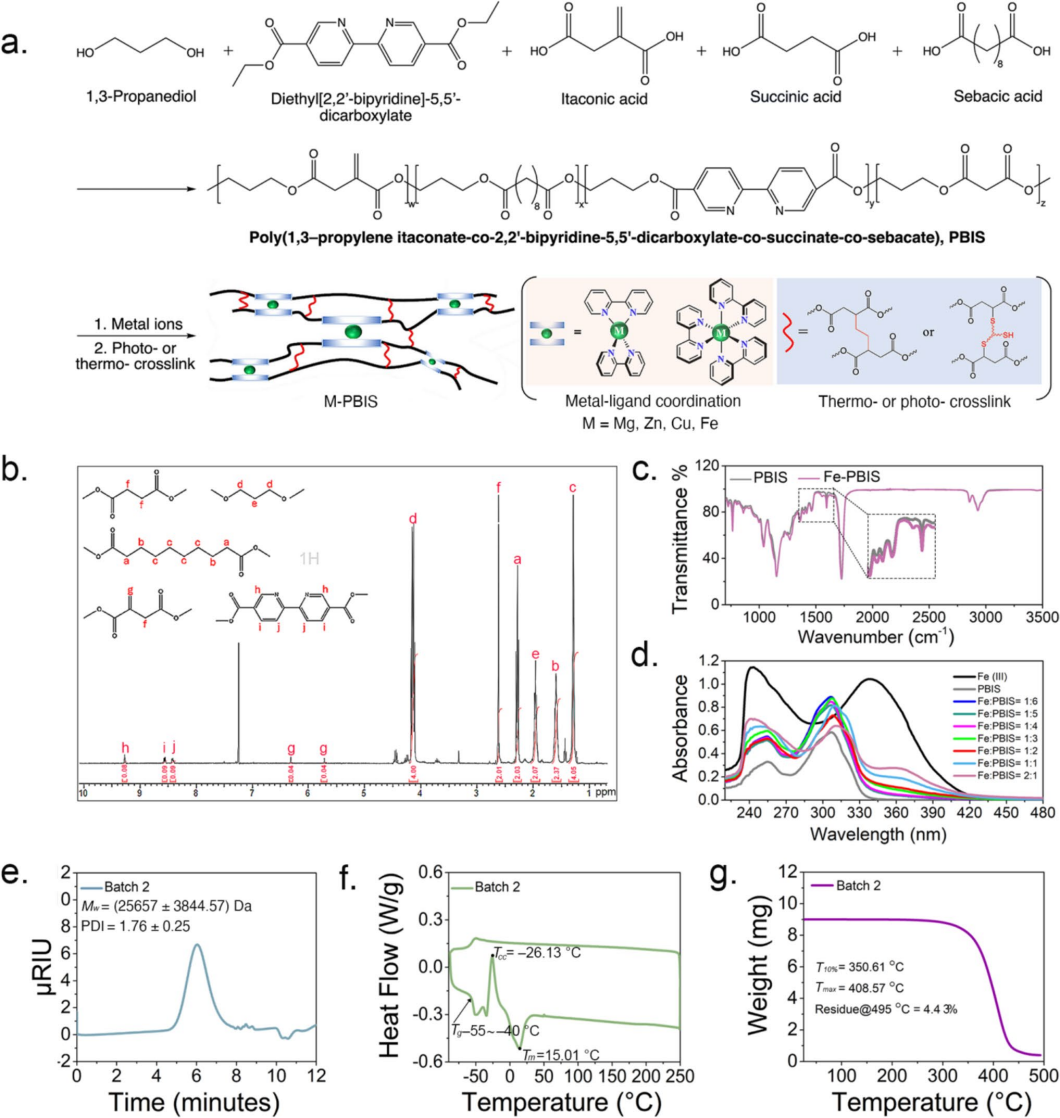
Design, synthesis, and physicochemical characterization of PBIS elastomers. **a** Step-growth polymerization scheme and dual-crosslinking strategy combining reversible metal–ligand coordination with covalent crosslinking. **b** Annotated ^1^H NMR spectrum of PBIS confirming monomer incorporation. **c** FTIR spectra of PBIS and Fe-PBIS confirming metal–ligand coordination. **d** UV–Vis absorption spectra of PBIS, Fe^3+^, and Fe-PBIS at varying Fe:PBIS ratios. **e** Molecular weight distribution by advanced polymer chromatography (APC). **f** Differential scanning calorimetry (DSC) thermogram. **g** Thermogravimetric analysis (TGA) curve. Data are presented as mean ± SD (*n* = 3)

PBIS elastomers were synthesized via a step-growth polyesterification strategy using diethyl [2,2′-bipyridine]-5,5′-dicarboxylate (Ethyl-DP), aliphatic diacids including succinic acid (SU) and sebacic acid (SA), itaconic acid (IA), and 1,3-propanediol (PDO) as the diol component (Fig. 1a). Polymerizations were conducted under anhydrous and oxygen-free conditions using titanium (IV) isopropoxide as the esterification catalyst and 4-methoxyphenol as a radical inhibitor (1 mol% relative to itaconic acid) to preserve the carbon–carbon double bonds. This approach enabled efficient formation of polyesters while maintaining the chemical integrity of both bipyridine ligands and vinyl functionalities. A key capability of this synthetic platform is its modular compositional design, which allows systematic and controllable tuning of (i) metal-coordination ligand density, (ii) backbone crystallinity and chain organization, and (iii) covalent crosslinkable site density. Metal-coordination ligand density was programmed by adjusting the molar fraction of Ethyl-DP relative to the total diacid content, while C = C crosslinkable site density was regulated by varying the molar fraction of itaconic acid, introducing vinyl groups for subsequent radical-mediated crosslinking [13]. Backbone crystallinity and mechanical response were modulated by varying the succinic-to-sebacic acid ratio, as sebacic acid promotes chain regularity and crystallization, whereas succinic acid disrupts crystalline packing [14]. Together, these parameters enable functionally complementary and compositionally programmed control over network architecture encoded at the polymer synthesis stage.

#### NMR Verification of Polymer Composition and Ligand Density

The successful incorporation of bipyridine ligands and itaconate units into the PBIS backbone was confirmed by ^1^H NMR spectroscopy. An annotated spectrum of PBIS (Batch 2) is shown in Fig. 1b, and the full spectra for Batches 1–7 are provided in Figs. S2–S8. The aromatic bipyridine signals (protons h, i, and j) and the itaconate vinyl signals (protons g) were well resolved from the aliphatic backbone resonances, enabling quantification of ligand incorporation. For all batches, integrals were normalized to the O-CH_2_ protons of the 1,3-propanediol unit (peak d), which served as the internal reference for composition analysis. The experimentally determined compositions are summarized in the inset tables in Figs. S2–S8. In all batches, the measured DP and IA contents were lower than the theoretical feed values, indicating incomplete incorporation of the DP and itaconate monomers during the step-growth polyesterification. For example, batches designed with 10 mol% IA showed actual IA contents of only 3.0–5.5 mol% (Batches 1–5), while Batch 6, designed with 5 mol% IA, exhibited an actual IA content of 2.0 mol%. Likewise, Batch 7, designed with 20 mol% IA, reached an actual IA content of 12.0 mol%, demonstrating that increasing IA in the feed increased IA incorporation, but not in a strictly proportional manner. Similar deviations were observed for DP incorporation with actual DP contents consistently below the theoretical values. These results indicate that the final polymer compositions deviated systematically from the feed ratios, likely due to differences in monomer reactivity, steric effects, and incorporation efficiency during polycondensation.

Accordingly, the experimentally measured NMR compositions, rather than the theoretical feed compositions, were used throughout this work to define bipyridine ligand density, itaconate content, and overall crosslinkable group density for subsequent structure–property analysis. This approach ensured that correlations between polymer composition, metal coordination, network formation, mechanical behavior, and biological performance were based on the actual chemical structures present in the synthesized materials. Collectively, these NMR results confirmed both successful incorporation of the bipyridine and itaconate functionalities and reproducible, batch-specific control of PBIS composition, providing a validated chemical basis for the subsequent coordination, processing, and performance studies.

#### Spectroscopic Evidence of Metal–Ligand Coordination

The coordination of metal ions with bipyridine ligands in PBIS was investigated using Fourier-transform infrared (FTIR) spectroscopy (Fig. 1c) and ultraviolet–visible (UV–Vis) absorption spectroscopy (Fig. 1d). Figure 1c compares the FTIR spectra of pristine PBIS (Batch 2) and Fe-PBIS. Both spectra retain the major features of the polyester backbone, including a strong ester carbonyl stretching band at ~ 1727 cm^−1^ in PBIS and ~ 1724 cm^−1^ in Fe-PBIS, aliphatic C–H-stretching bands near ~ 2855 and 2928–2929 cm^−1^, and ester C–O–C-stretching bands in the 1150–1270 cm^−1^ region, indicating that the polymer backbone remains structurally intact after Fe^3+^ incorporation. Upon coordination, subtle but reproducible changes were observed in the bipyridine-associated vibrational region (1350–1650 cm^−1^), as magnified in the inset. In the 1588–1598 cm^−1^ region, assigned to aromatic C = N and C = C-stretching vibrations of the bipyridine moiety [15], Fe-PBIS exhibited a clear increase in absorption intensity relative to PBIS, with transmittance decreasing from approximately 91.3% to 84.5% at ~ 1594 cm^−1^, while showing minimal peak shift. This behavior is consistent with coordination-induced perturbation of the bipyridine electronic environment upon Fe^3+^ binding to the pyridine nitrogen atoms [16]. Additional intensity changes were also observed in the 1360–1460 cm^−1^ region, including bands near 1391–1392 cm^−1^, which are attributed to bipyridine ring deformation modes and further support ligand–metal interaction [17]. A slight shift of the ester carbonyl absorption from ~ 1727 cm^−1^ in PBIS to ~ 1724 cm^−1^ in Fe-PBIS was observed in the raw spectra, although the difference is subtle in the overlaid plot. This small change may reflect minor electronic effects transmitted through the polymer backbone upon Fe^3+^-bipyridine coordination. In contrast, the aliphatic C–H-stretching bands and the ester C–O–C bands remained largely unchanged, indicating that Fe^3+^ coordination occurs primarily at the bipyridine nitrogen sites without disrupting the polyester backbone. Collectively, the FTIR data support Fe^3+^-bipyridine coordination while preserving the structural integrity of the polymer matrix.

UV–Vis absorption spectroscopy provided additional evidence for metal–ligand coordination and enabled optimization of the Fe:PBIS ratio (Fig. 1d). Free Fe^3+^ in solution exhibited a strong absorption peak at approximately 243 nm and a broad band extending from approximately 280 to 420 nm with a maximum at approximately 338 nm, characteristic of hydrated Fe^3+^ species. Pristine PBIS (Batch 2) displayed two distinct absorption peaks at approximately 255 nm and approximately 307 nm, assigned to ligand-centered π → π transitions of the bipyridine chromophore [18], with negligible absorption above 350 nm. Upon mixing Fe^3+^ with PBIS across a range of Fe:PBIS molar ratios, the broad Fe^3+^ band in the 280–420 nm region was substantially suppressed, while the bipyridine-associated peaks at approximately 255 nm and ~approximately 307 nm became dominant, supporting Fe^3+^-bipyridine complex formation. At high Fe^3+^ loadings (Fe:PBIS = 2:1 and 1:1), residual absorption in the 350–420 nm region was still observed, suggesting the presence of excess or less efficiently coordinated Fe^3+^ beyond the available bipyridine-binding capacity [19]. As the Fe:PBIS ratio decreased from 1:2 to 1:6, this residual long-wavelength absorption progressively diminished, and the spectra at Fe:PBIS ratios of 1:4 to 1:6 showed minimal absorbance above 350 nm, consistent with more complete coordination of Fe^3+^ by the available bipyridine ligands. The spectral profiles also converged in this lower-Fe regime, indicating that the accessible bipyridine coordination sites approached saturation. Based on this titration, a Fe:PBIS ratio of approximately 1:3 was selected as the working formulation, as it provided strong coordination-associated absorption while minimizing free Fe^3+^. This trend is broadly consistent with tris(bipyridine)-like Fe coordination environments [20], yielding a well-defined metal-crosslinked PBIS network.

### Molecular Weight and Thermal Properties

The molecular weight and distribution of PBIS polymers were characterized by advanced polymer chromatography (APC) to evaluate polymerization efficiency and batch-to-batch reproducibility (Fig. 1e and Fig. S9). The representative Batch 2 polymer (Fig. 1e) exhibited a weight-average molecular weight (*M*_*w*_) of (25657 ± 3845) Da and a PDI of 1.76 ± 0.25 (*n* = 4). Across Batches 1–7 (Fig. 1e, Fig. S9), APC traces showed a dominant primary polymer peak accompanied, in most batches, by a minor lower-molecular-weight secondary peak/shoulder, consistent with the presence of small amounts of oligomeric species. While modest batch-to-batch differences in *M*_*w*_ and dispersity were observed, with *M*_*w*_ ranging from (16702 ± 1450) Da to (26244 ± 3406) Da and PDI from 1.62 ± 0.18 to 1.91 ± 0.16, the overall distributions were comparable and within the expected range for functional step-growth polyesters incorporating aromatic ligands and mixed aliphatic diacid segments. Reported *M*_*w*_ and PDI values were determined from the principal polymer peak only, excluding the minor low-molecular-weight fraction.

Thermal transitions of PBIS polymers were investigated by differential scanning calorimetry (DSC) to evaluate composition-dependent thermal behavior and crystallization tendency (Fig. 1f and Fig. S10). Batch 2 (Fig. 1f) exhibited a broad glass-transition region spanning approximately –55 °C to –40 °C, followed by cold crystallization at Tcc = –26.13 °C and melting at Tm = 15.01 °C. The presence of a cold-crystallization peak indicates that part of the polymer remained amorphous during cooling and subsequently reorganized into crystalline domains upon reheating, consistent with intermediate crystallization kinetics in functional copolyesters [21]. Across the batch series (Fig. S10), the thermal transitions were strongly dependent on diacid composition and functional comonomer content. Batch 1 (feed: 80 mol% SA, 0 mol% SU) showed the highest crystallization and melting temperatures, with a sharp exothermic crystallization peak at 16.18 °C and a melting transition at 49.63 °C, consistent with enhanced chain regularity and packing efficiency arising from the longer sebacic acid segments [22]. Compared with the other batches, the crystallization event in Batch 1 occurred at a substantially higher temperature, reflecting faster crystallization kinetics and greater intrinsic crystallizability of the sebacic-acid-rich polymer. Although a distinct *T*_*g*_ was not clearly resolved for Batch 1, this likely reflects its relatively high crystalline fraction and correspondingly reduced amorphous phase. In contrast, Batch 3 (feed: 80 mol% SU, 0 mol% SA) exhibited predominantly amorphous behavior without a clearly resolved melting transition, suggesting reduced crystallization propensity associated with the shorter succinic acid segments, which limit formation of extended crystallizable sequences [23]. Batches 2, 4, 5, 6, and 7, all containing approximately balanced SA/SU ratios, displayed semicrystalline behavior characterized by cold crystallization and low-temperature melting transitions. Within this subset, variation in bipyridine (DP) and itaconate (IA) content further modulated the transition temperatures. Batch 4 (low DP feed, 5 mol%) exhibited *T*_cc_ = –28.53 °C and *T*_m_ = 16.23 °C, whereas Batch 5 (high DP feed, 20 mol%) showed *T*_cc_ = –27.3 °C and *T*_m_ = 17.11 °C, indicating only modest changes with increasing DP content. Likewise, Batch 6 (low IA feed, 5 mol%) displayed *T*_cc_ = –28.15 °C and *T*_m_ = 17.83 °C, whereas Batch 7 (high IA feed, 20 mol%) showed a cold-crystallization peak shifted to a higher temperature (*T*_cc_ = –18.21 °C) together with a substantially reduced melting temperature (*T*_m_ = 4.63 °C), indicating altered crystallization kinetics and reduced crystal stability at elevated itaconate content. Relative to the SA-rich Batch 1 polymer, the balanced SA/SU formulations exhibited markedly lower crystallization and melting temperatures [14], consistent with statistical incorporation of C4 and C10 diacid units that disrupts chain regularity, lowers packing efficiency, and reduces crystal stability. Overall, increasing succinic acid and functional comonomer content yielded more intermediate and tunable thermal behavior.

Thermal stability was further evaluated by thermogravimetric analysis (TGA) (Fig. 1g and Fig. S11). Batch 2 exhibited a *T*_*10%*_ of 350.6 °C, *T*_*max*_ of 408.6 °C, and 4.4% residual mass at 495 °C (Fig. 1g). Across Batches 1–7, the polymers showed broadly consistent thermal degradation behavior, with *T*_*10%*_ values ranging from 313.3 to 371.7 °C and *T*_*max*_ values ranging from 372.1 to 408.8 °C (Fig. S11), indicating good thermal stability across independent syntheses. Residual mass at 495 °C varied from 3.8% to 8.5% among batches, consistent with modest char formation during decomposition of the aromatic bipyridine-containing polyester structure [24]. Together, APC, DSC, and TGA results across Batches 1–7 (Fig. 1e–g and Fig. S9–S11) demonstrate reproducible molecular weight control, consistent thermal transition behavior, and high thermal stability. These properties establish a robust and reliable PBIS materials foundation for subsequent metal coordination, dual crosslinking, and scaffold fabrication described in the following sections.

### Compositional Effects on Polymer Flexibility and Network Formation

**Fig. 2:**
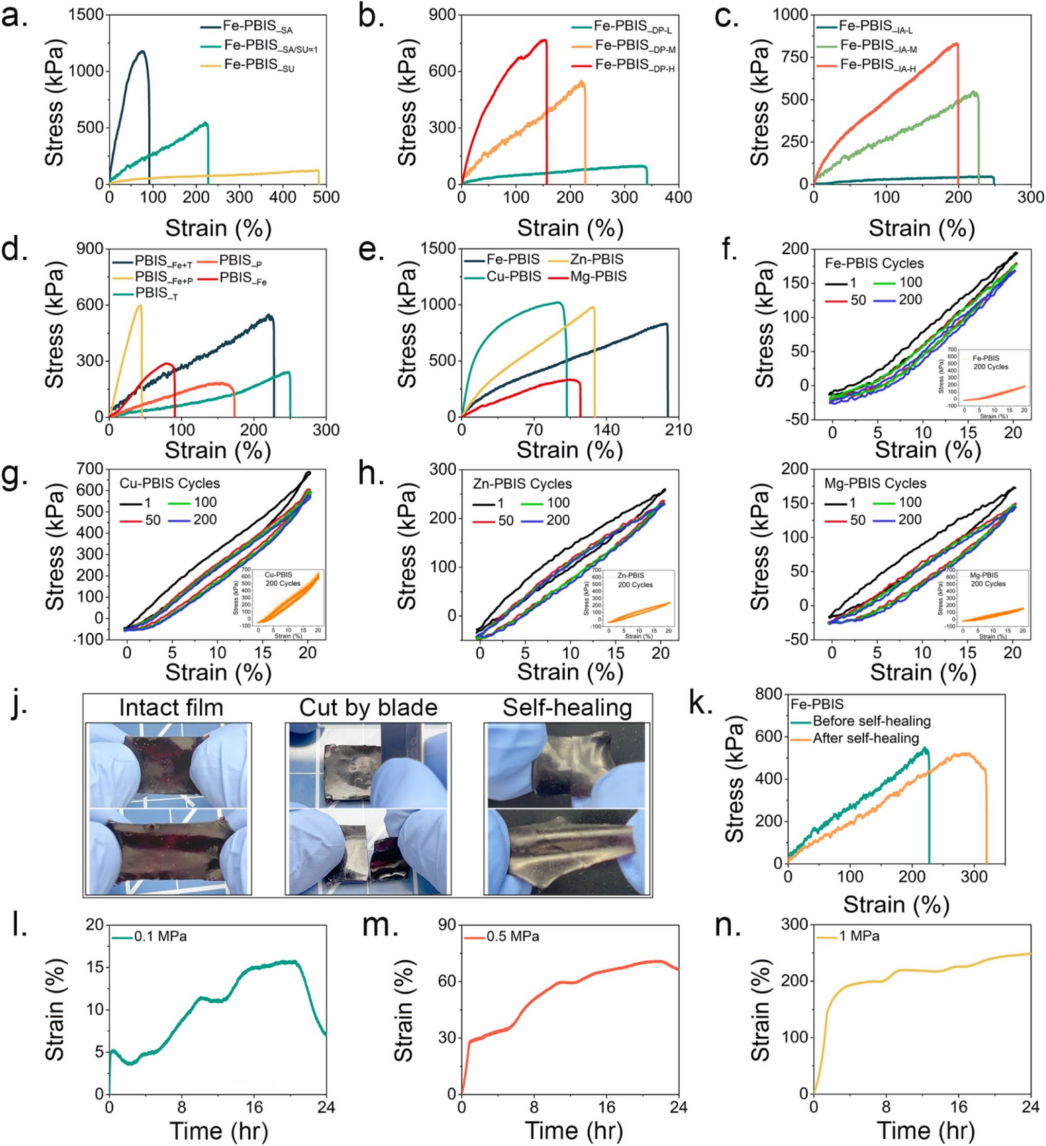
Mechanical tuning and adaptive behavior of dual-crosslinked PBIS elastomers. Tensile stress–strain curves showing the effect of **a** diacid composition, **b** bipyridine ligand density, and **c** itaconate content. **d** Tensile curves comparing coordination-only, covalent-only, and dual-crosslinked networks. **e** Tensile curves for PBIS coordinated with different metal ions. **f**–**i** Cyclic loading–unloading curves over 200 cycles at 20% strain for Fe-, Cu-, Zn-, and Mg-PBIS, respectively. **j** Photographs of macroscopic self-healing using a cut-and-rejoin protocol. **k** Tensile curves before and after self-healing. **l**–**n** Creep strain profiles at 37 °C under constant stresses of 0.1, 0.5, and 1 MPa, respectively. Data are presented as mean ± SD (*n* = 4)

Fe-PBIS was selected as a representative M-PBIS system to examine the compositional effects on polymer flexibility and network formation in Fig. 2a–c. All samples discussed in this section are, therefore, Fe-coordinated, thermally crosslinked networks. The sample codes used in this section follow the nomenclature defined in Fig. S1. PBIS__SA_ and PBIS__SU_ denote the SA-only and SU-only diacid formulations, respectively. PBIS__SA/SU=1_ denotes the equimolar SA/SU formulation. PBIS__DP-L_, PBIS__DP-M_, and PBIS__DP-H_ denote the low-, medium-, and high-bipyridine formulations, respectively. PBIS__IA-L_, PBIS__IA-M_, and PBIS__IA-H_ denote the low-, medium-, and high-itaconate formulations, respectively.

#### Role of Diacid Composition (SU/SA Ratio)

The choice of diacid monomer ratio (SA/SU) had a profound impact on the mechanical properties of the Fe-PBIS networks. As shown in Fig. 2a, Fe-PBIS__SA_ exhibited high initial stiffness, reaching an ultimate tensile stress (UTS) of (1202.51 ± 138.02) kPa at a strain at fracture of 91.45% ± 14.51% (Fig. S12). This behavior directly correlates with the pronounced crystallinity observed in SA-rich polymers by DSC (Fig. S10), where the long C10 aliphatic spacer promotes chain regularity and packing, restricting chain mobility even above the melting temperature [25]. In contrast, Fe-PBIS__SU_ demonstrated dramatically different behavior, with exceptional extensibility at a strain at fracture of (491.04% ± 49.10%), significantly reduced strength at a UTS of (175.22 ± 50.50) kPa, and a low Young’s modulus of (107.14 ± 21.11) kPa, nearly 20-fold lower than that of Fe-PBIS__SA_ at (2143.89 ± 409.56) kPa. This high compliance is consistent with the predominantly amorphous character of SU-rich polymers, which showed no resolvable crystallization events in DSC analysis, reflecting inefficient chain packing and enhanced segmental mobility [14]. The intermediate composition Fe-PBIS__SA/SU=1_ exhibited behavior bridging these extremes, with a strain at fracture of 205.81% ± 28.67%, UTS of (530.45 ± 63.82) kPa, and Young’s modulus of (330.21 ± 25.69) kPa. This response is consistent with the semicrystalline thermal behavior observed for balanced SA/SU compositions, where mixed C4/C10 diacid incorporation suppresses long-range order while retaining limited crystallization. As a result, these networks likely contain mechanically distinct regions associated with crystalline and amorphous chain segments, enabling efficient stress transfer without severely constraining chain deformation. Toughness values further illustrated these structure–property relationships: Fe-PBIS__SA_ achieved the highest toughness of (718.10 ± 69.78) kJ m^−3^ through combined strength and moderate ductility, while Fe-PBIS__SU_ showed comparable toughness of (527.41 ± 169.64) kJ m^−3^ despite lower strength. These results demonstrate that diacid composition provides tunable control over mechanical properties from stiff, crystalline networks to soft, highly deformable elastomers.

#### Effect of Bipyridine Ligand Density

Bipyridine ligand density directly influences the extent of metal–ligand coordination and thus the density of coordination-mediated junctions within the PBIS network. As shown in Fig. 2b and quantified in Fig. S13, increasing ligand content (PBIS__DP-L_ → PBIS__DP-M_ → PBIS__DP-H_) led to substantial enhancements in mechanical strength and stiffness. Fe-PBIS__DP-H_, containing the highest bipyridine content, exhibited the greatest UTS of (832.68 ± 70.97) kPa and Young’s modulus of (1233.85 ± 364.07) kPa, reflecting dense coordination crosslinking that restricts chain mobility and increases load-bearing capacity. However, this increase in stiffness was accompanied by reduced extensibility, with strain at fracture decreasing to 142.51% ± 53.76%. In contrast, Fe-PBIS__DP-L_, with the lowest ligand density, demonstrated the highest elongation at break of (331.65% ± 57.67% but minimal mechanical strength, with a UTS of (95.62 ± 20.82) kPa and a Young’s modulus of (71.26 ± 27.67) kPa. The sparse coordination sites allow greater chain mobility and deformation but provide insufficient crosslink density to effectively transmit stress across the network. The intermediate composition, Fe-PBIS__DP-M_, achieved a balanced mechanical response with moderate extensibility of 205.81% ± 28.67%, a UTS of (530.45 ± 63.82) kPa, and a Young’s modulus of (330.21 ± 25.69) kPa. Notably, Fe-PBIS__DP-M_ exhibited optimal toughness of (602.98 ± 91.56) kJ m^−3^, suggesting an optimal coordination density that balances energy dissipation through chain deformation with sufficient network connectivity to prevent premature failure. Together, these results highlight the dual role of bipyridine units as both mechanical reinforcements and coordination sites, where excessive coordination over-constrains chain motion and limits elastic deformation. Ligand density, therefore, provides a powerful and orthogonal design parameter for tuning coordination strength and network stiffness independently of backbone chemistry or covalent crosslinking [26].

#### Effect of Itaconate Content

Similar to bipyridine ligand density, itaconate content modulates the mechanical response of Fe-PBIS networks by increasing effective network connectivity. However, unlike bipyridine units that introduce reversible coordination junctions, itaconate groups primarily contribute permanent covalent crosslinks upon network formation, while also influencing backbone structure through diacid substitution [27]. As shown in Fig. 2c and Fig. S14, increasing itaconate incorporation (PBIS__IA-L_ → PBIS__IA-M_ → PBIS__IA-H_) led to systematic increases in stiffness, strength, and toughness, with comparatively minor effects on extensibility. Fe-PBIS__IA-L_ exhibited a highly compliant response with a UTS of (49.05 ± 7.64) kPa and modulus of (59.87 ± 36.23) kPa, reflecting insufficient covalent reinforcement despite substantial extensibility. Increasing itaconate content to intermediate and high levels resulted in pronounced mechanical reinforcement, with UTS rising to (530.45 ± 63.82) kPa (Fe-PBIS__IA-M_) and (959.60 ± 335.48) kPa (Fe-PBIS__IA-H_), accompanied by corresponding increases in Young’s modulus (Fig. S14). Notably, strain at fracture remained relatively constant across the series (approximately 200%–225%), indicating that covalent crosslinking via itaconate enhances load-bearing capacity without severely restricting chain deformation. As a result, toughness increased monotonically with itaconate content, reaching (1338.97 ± 663.75) kJ m^−3^ for Fe-PBIS__IA-H_. These results demonstrate that itaconate incorporation provides a functionally complementary mechanism to coordination density for reinforcing Fe-PBIS networks, enabling simultaneous enhancement of stiffness, strength, and energy dissipation within the studied compositional window.

### Single- Versus Dual-Crosslinking Strategies in PBIS Networks

The mechanical consequences of different crosslinking strategies in PBIS (Batch 2) elastomers were systematically evaluated by comparing coordination-only, covalent-only, and dual-crosslinked networks prepared from the same polymer composition (Fig. 2d and Fig. S15). This controlled comparison isolates the role of crosslinking chemistry in governing elastic response, extensibility, and mechanical stability. Here, PBIS__Fe_ denotes Fe-coordinated PBIS without additional covalent crosslinking; PBIS__T_ and PBIS__P_ denote thermally and photocrosslinked PBIS without Fe coordination, respectively. PBIS__Fe+T_ and PBIS__Fe+P_ denote Fe-coordinated PBIS combined with thermal and photocrosslinking, respectively. PBIS networks relying solely on metal–ligand coordination (PBIS__Fe_) exhibited limited extensibility, with strain at fracture of 89.95% ± 16.62%, relatively low mechanical strength with a UTS of (279.56 ± 51.10) kPa and a Young’s modulus of (404.38 ± 119.18) kPa. While Fe-bipyridine coordination provides reversible physical junctions that contribute to network connectivity and enable some elastic recovery, these dynamic interactions alone were insufficient to prevent stress relaxation and permanent deformation under sustained load, resulting in modest toughness of (152.05 ± 46.04) kJ m^−3^.

PBIS networks crosslinked exclusively through covalent bonds showed contrasting mechanical behavior depending on the crosslinking mode. PBIS__T_, prepared by thermally initiated radical crosslinking using 4,4′-azobis(4-cyanovaleric acid) (ACVA), exhibited high extensibility, with a strain at fracture of 270.77% ± 63.26% but very low stiffness, with a Young’s modulus of (91.07 ± 8.50) kPa and strength, with a UTS of (219.28 ± 38.95) kPa. This behavior suggests a relatively low effective crosslink density, in which covalent junctions provide network integrity while permitting substantial chain mobility, resulting in moderate toughness of (271.66 ± 126.20) kJ m^−3^ [28]. In contrast, PBIS__P_, crosslinked via photo-initiated thiol-ene chemistry using trimethylolpropane tris(3-mercaptopropionate) (TMMP) and lithium phenyl-2,4,6-trimethylbenzoylphosphinate (LAP) [29], displayed reduced extensibility of 136.70% ± 32.38%, with a UTS of (243.12 ± 75.75) kPa and a Young’s modulus of (294.35 ± 142.95) kPa. Compared with PBIS__T_, the lower toughness of (174.61 ± 29.42) kJ m^−3^ indicates a more rigid, less energy-dissipative network, consistent with a higher density of permanent covalent junctions formed during photocrosslinking.

Notably, PBIS elastomers incorporating both metal–ligand coordination and covalent crosslinking exhibited synergistic mechanical performance. The dual-crosslinked network combining Fe coordination with thermal crosslinking (PBIS__Fe+T_) achieved balanced properties, with a strain at fracture of 205.81% ± 28.67%, UTS of (530.45 ± 63.82) kPa, Young’s modulus of (330.21 ± 25.69) kPa, and the highest toughness of (602.98 ± 91.56) kJ m^−3^. This synergistic mechanical response is attributable to the complementary roles of coordination and covalent crosslinks, in which dynamic metal–ligand interactions promote stress redistribution and energy dissipation [30], while permanent covalent junctions maintain network integrity and resist deformation under load [31]. In contrast, the combination of Fe coordination with photo-initiated thiol-ene crosslinking (PBIS__Fe+P_) is chemically more complex, because thiol compounds can compete with bipyridine ligands for Fe coordination and may reduce Fe^3+^ to Fe^2+^ with concomitant oxidation of thiols to disulfides, potentially compromising the intended dual-crosslink architecture [32]. Consistent with this possibility, PBIS__Fe+P_ exhibited dramatically different behavior from PBIS__Fe+T_, showing exceptionally high Young’s modulus of (1427.97 ± 371.78) kPa and good strength, with a UTS of (560.78 ± 113.26) kPa, but severely reduced extensibility of 53.12% ± 7.34% and low toughness of (176.70 ± 49.04) kJ m^−3^. This unusually rigid and brittle response, more consistent with a densely covalently crosslinked network than a synergistic dual-crosslinked one, may partly reflect disruption of Fe-bipyridine coordination by the thiol reagent rather than true cooperative dual crosslinking. Together, these results demonstrate that dual crosslinking is most effective when the two crosslinking modes are chemically compatible. Among the strategies evaluated, PBIS__Fe+T_ emerged as the most effective dual-crosslinking approach, combining the energy-dissipative capacity of reversible Fe-bipyridine coordination with the structural reinforcement of thermally initiated covalent crosslinks.

### Tuning Crosslinking Behavior via Metal Chemistry

#### Effect of Metal Ion Identity on Tensile Mechanical Behavior

Beyond compositional tuning of the polymer backbone, the choice of metal ion provides an additional mechanism to modulate mechanical properties through differences in coordination strength and network connectivity [11]. To isolate the effect of metal ion identity, a single PBIS formulation (Batch 7) was used for all samples in this comparison and crosslinked with different metal ions (Fe^3+^, Cu^2+^, Zn^2+^, and Mg^2+^) (Fig. 2e and Fig. S16). Batch 7 was selected, because its elevated itaconate content provides a stronger covalent crosslinking background, enabling metal-ion-dependent effects on network mechanics to be evaluated within a common polymer formulation. The stress–strain responses revealed pronounced metal-dependent mechanical behavior. Cu-PBIS exhibited the highest stiffness, with a Young’s modulus of (3180.79 ± 103.07) kPa and strength, with a UTS of (1013.02 ± 34.79) kPa, but limited extensibility, with a strain at fracture of 90.86% ± 11.14%, resulting in a relatively stiff and less deformable network with moderate toughness of (708.21 ± 113.14) kJ m^−3^. Zn-PBIS showed comparable strength, with a UTS of (1002.67 ± 103.46) kPa with slightly increased extensibility of 118.71% ± 10.57% and similar toughness of (689.84 ± 112.00) kJ m^−3^, indicating a balance between stiffness and deformability. In contrast, Fe-PBIS demonstrated substantially higher extensibility of 224.35% ± 62.34% while maintaining competitive strength, with a UTS of (959.60 ± 335.48) kPa, and moderate stiffness, with a Young’s modulus of (1037.39 ± 302.10) kPa. Notably, Fe-PBIS achieved the highest toughness of (1338.97 ± 663.75) kJ m^−3^, nearly double that of Cu- and Zn-coordinated networks. This behavior suggests that Fe^3+^ coordination enables a favorable combination of mechanical reinforcement and deformation tolerance, allowing efficient energy dissipation under tensile loading [33]. Mg-PBIS exhibited the most compliant response, with the lowest Young’s modulus of (560.99 ± 87.86) kPa, reduced strength, with a UTS of (342.99 ± 12.66) kPa, and minimal toughness of (222.76 ± 30.59) kJ m^−3^, consistent with weaker metal–ligand interactions providing limited effective network reinforcement [34]. Collectively, these results demonstrate that metal ion identity serves as a powerful design parameter for tuning PBIS network mechanics, spanning from stiff, high-modulus elastomers to highly extensible, tough networks.

#### Cyclic Mechanical Behavior and Energy Dissipation

The cyclic mechanical behavior and energy dissipation of the same metal-ion series based on Batch 7 were further evaluated by cyclic tensile testing at 20% strain to assess mechanical recovery, hysteresis, and resistance to repeated loading [35] (Fig. 2f–i). These experiments probe how the common covalent network background, together with metal-dependent coordination interactions, governs repeated deformation and energy dissipation. Among the four systems, Cu-PBIS exhibited the highest peak stresses and the largest hysteresis loop areas during cyclic loading, indicating the greatest absolute energy dissipation per cycle (Fig. 2f–i). Fe-PBIS, Zn-PBIS, and Mg-PBIS exhibited broadly similar cyclic mechanical responses, characterized by stable stress–strain behavior over 200 loading–unloading cycles and relatively small hysteresis loops. All four systems maintained consistent peak stresses throughout cycling, indicating good mechanical stability and minimal accumulation of permanent deformation. The narrow hysteresis loops observed for Fe-PBIS, Zn-PBIS, and Mg-PBIS suggest predominantly elastic-dominated deformation with limited viscoelastic energy loss per cycle, consistent with a robust covalently crosslinked network in which metal–ligand coordination further modulates the stress response [36]. Within this group, Fe-PBIS displayed the smallest loop area, whereas Zn-PBIS and Mg-PBIS showed similarly small hysteresis loops. Zn-PBIS sustained higher stresses than Fe-PBIS and Mg-PBIS, while Mg-PBIS maintained a comparatively low-stress but still stable cyclic response. The observation that all four formulations maintained stable cyclic behavior suggests that the common covalent network provides the dominant contribution to elastic recovery and mechanical stability under repeated loading, while metal-ion identity primarily tunes stress level and hysteresis through differences in coordination strength.

Compared with Fe-PBIS, Zn-PBIS, and Mg-PBIS, the substantially larger loop area of Cu-PBIS indicates greater absolute hysteretic energy dissipation, consistent with its much higher stress response under the same applied strain. Together, these results suggest that the covalent crosslinking background plays the dominant role in maintaining elastic network integrity under cyclic loading, whereas metal-ion identity primarily modulates stiffness, stress level, and the extent of hysteretic energy dissipation. Within this framework, Cu^2+^ provides the highest stress-bearing capacity and greatest energy dissipation, Fe^3+^ yields the most elastic response with the smallest hysteresis loop, and Zn^2+^ and Mg^2+^ show similarly small loop areas with intermediate and lower stress levels, respectively. This distinction highlights metal ion identity as a key design parameter for tuning cyclic energy dissipation and fatigue behavior in PBIS elastomers, enabling selection of coordination chemistry based on application-specific demands for elasticity versus damping.

### Time-Dependent Mechanical Behavior Enabled by Coordination-Mediated Networks

#### Self-Healing Behavior Under Mechanical Damage

The ability of damaged PBIS (Batch 7) elastomers to restore interfacial connectivity was evaluated through macroscopic self-healing experiments (Fig. 2j–k, Fig. S17). Fe-PBIS films were completely severed into two separate pieces using a blade, and the cut surfaces were then brought back into contact and incubated at 37 °C under controlled benchtop conditions. A 200 g weight was applied across the reconnected interface to maintain intimate contact between the cut surfaces during healing. After 1 h, the rejoined samples regained sufficient mechanical integrity to be handled and subjected to tensile testing. Quantitative tensile measurements revealed substantial recovery of mechanical performance following healing (Fig. 2k and Fig. S17). The healed sample exhibited a stress–strain curve similar in shape to that of the pristine material, with ultimate tensile stress remaining essentially unchanged, at (530.45 ± 63.82) kPa before healing vs. (549.71 ± 72.64) kPa after healing. The healed sample also showed increased strain at fracture of 287.60% ± 40.51% vs. 205.81% ± 28.67% for the pristine sample, together with a modest decrease in Young’s modulus from (330.21 ± 25.69) kPa to (248.38 ± 21.35) kPa and an increase in toughness from (602.98 ± 91.56) kJ m^−3^ to (830.24 ± 112.57) kJ m^−3^. These changes suggest that interfacial healing is accompanied by network rearrangement and redistribution of load-bearing pathways, enabling recovery of stress transfer while increasing deformability and energy dissipation [37].

Because permanent covalent crosslinks cannot readily reform once fractured, the observed interfacial healing and mechanical recovery indicate that reversible metal–ligand coordination interactions play a major role in re-establishing connectivity across the damaged interface. The dynamic nature of Fe^3+^-bipyridine coordination likely enables polymer chains to reorganize and form new coordination bonds across the cut surfaces, partially restoring mechanical continuity between the severed pieces [38]. The increased extensibility and toughness after healing further suggest that the reorganized network accommodates deformation more efficiently, with improved stress redistribution and dissipation compared with the as-prepared material [39]. This experiment provides a proof-of-concept that Fe-coordinated PBIS networks can recover substantial interfacial mechanical integrity under mild healing conditions, highlighting the functional advantage of incorporating reversible coordination crosslinks alongside permanent covalent bonds.

#### Creep Behavior Under Sustained Mechanical Loading

Time-dependent deformation of Fe-PBIS (Batch 7) elastomers was investigated through creep testing under constant uniaxial tensile stress at physiological temperature (37 °C) (Fig. 2l-n). To span the range of physiological stresses encountered in soft-tissue applications, applied stresses of 0.1 MPa, 0.5 MPa, and 1 MPa were selected to represent low, moderate, and high mechanical loading conditions relevant to vascular and other soft human tissue engineering applications [40]. Under all loading conditions, Fe-PBIS exhibited stress-dependent viscoelastic creep behavior characterized by an initial rapid strain increase followed by a slower, time-dependent strain evolution over a 24 h period. At the lowest applied stress (0.1 MPa), Fe-PBIS showed limited deformation, with strain gradually increasing to approximately 15% over 24 h (Fig. 2l). The absence of continuous strain acceleration indicates effective resistance to long-term flow under low physiological stresses. At an intermediate stress of 0.5 MPa, the material exhibited increased creep, reaching approximately 70% strain over 24 h, with a pronounced primary creep regime followed by a slower secondary creep response (Fig. 2m). When subjected to the highest stress level (1 MPa), Fe-PBIS underwent substantial deformation, exceeding 200% strain (Fig. 2n). However, strain evolution remained progressive and bounded, with no evidence of runaway deformation or catastrophic failure over the test duration. The stress-dependent creep response reflects the hybrid nature of the Fe-PBIS network. Reversible metal–ligand coordination junctions permit time-dependent chain rearrangement and stress redistribution under sustained load, giving rise to the observed creep behavior [41]. Simultaneously, permanent covalent crosslinks preserve global network connectivity, preventing uncontrolled deformation and mechanical failure even under elevated stresses [42]. This combination enables Fe-PBIS elastomers to accommodate prolonged mechanical loading while maintaining structural integrity, a key requirement for load-bearing biomedical applications that experience continuous and fluctuating physiological stresses. The slight decrease in strain observed after approximately 20 h under 0.1 and 0.5 MPa loading was reproducible across multiple independent experiments. While minor load-control fluctuations inherent to long-duration creep testing cannot be fully excluded, particularly at low-stress levels where total creep strains are relatively small, the reproducibility of this response suggests that it may at least partly reflect delayed elastic recovery arising from the permanent covalent crosslink network, whereby the elastic restoring force progressively dominates as dynamic Fe^3+^–bipyridine coordination bonds redistribute over extended time. This behavior is absent at 1 MPa, where the applied stress continuously drives chain extension beyond the recovery capacity of the covalent network, consistent with the stress-dependent transition from partial viscoelastic recovery to progressive creep observed across the loading series.

### Rheological Behavior and Processability of PBIS Precursor and Networks

#### Rheological Characterization of PBIS Precursor

**Fig. 3:**
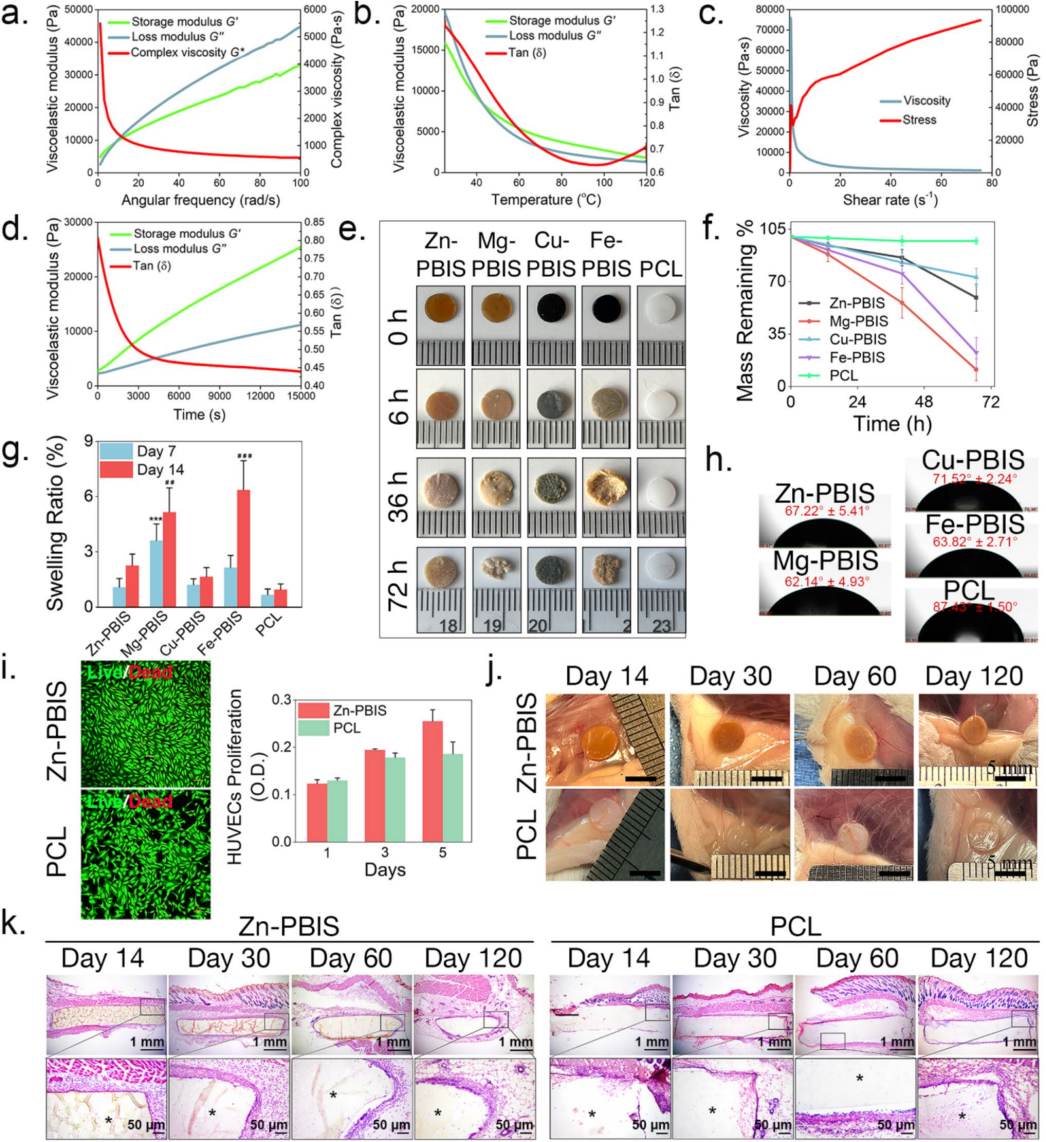
Rheological behavior, degradation, surface properties, and biological performance of PBIS elastomers. **a**–**c** Rheological characterization of PBIS precursor, including frequency sweep at 37 °C, temperature sweep from 25 to 120 °C, and steady-shear viscosity. **d** Time sweep monitoring thermal crosslinking kinetics of Fe-PBIS at 120 °C. **e**–**g** Representative images, mass remaining, and swelling ratio of M-PBIS and PCL films after accelerated alkaline degradation. **h** Water contact angles of M-PBIS and PCL. **i** Live/dead staining and HUVEC proliferation on Zn-PBIS and PCL. **j**, **k** Gross images and H&E-stained sections of Zn-PBIS and PCL after subcutaneous implantation for up to 120 days. Data are mean ± SD. Statistical significance: *p < 0.05, ** p < 0.01, *** p < 0.001

The rheological properties of the PBIS precursor (Batch 7) were systematically characterized to define the processing window and flow behavior of the polymer precursor prior to crosslinking (Fig. 3a-c). Frequency sweep measurements at 37 °C showed that *G′* was slightly higher than *G″* at the lowest angular frequencies, followed by a crossover in the low-frequency regime after which *G″* exceeded *G′* over most of the measured range (Fig. 3a) [43]. With increasing frequency, both moduli increased, and a crossover between *G′* and *G″* was observed at approximately 10–15 rad s^−1^, defining a characteristic relaxation timescale for the uncrosslinked PBIS chains. Above this frequency, *G″* exceeded *G′*, reflecting a transition toward viscous-dominated behavior as chain relaxation could no longer keep pace with the applied deformation rate [44]. The complex viscosity (η*) exhibited pronounced shear-thinning behavior, decreasing by nearly an order of magnitude with increasing frequency, demonstrating favorable flow characteristics for processing operations [45].

Temperature-dependent rheological behavior of the PBIS precursor was evaluated by temperature sweep measurements from 25 to 120 °C to assess thermal stability and define processing limits prior to crosslinking (Fig. 3b). With increasing temperature, both the storage modulus (*G′*) and loss modulus (*G″*) decreased monotonically, reflecting enhanced polymer chain mobility and progressive reduction in entanglement density as thermal energy increased [46]. At room temperature (25 °C), the PBIS precursor exhibited viscous-dominated behavior, with *G″* (≈ 19000 Pa) exceeding *G′* (≈ 16000 Pa), consistent with melt-like viscoelastic response favorable for processing. As temperature increased, a crossover between *G′* and *G″* was observed near 43 °C at approximately 8500 Pa, marking a transition in viscoelastic balance. Above this temperature, *G′* slightly exceeded *G″*, while both moduli continued to decrease, indicating an increased elastic contribution within an overall softening, highly deformable material state. With further heating to 120 °C, both moduli decreased to the low-kPa range (*G′* ≈ 2000 –2500 Pa; *G″* ≈ 1000 –1500 Pa), demonstrating continuous softening and increased flowability without evidence of modulus plateaus or abrupt increases [47]. The damping factor, tan(*δ*), decreased from approximately 1.2 at 25 °C to a minimum of approximately 0.63 near 96 °C, reflecting a gradual shift in the balance between viscous and elastic contributions rather than the emergence of solid-like behavior. A slight increase in tan(*δ*) at the highest temperatures suggests increased viscous dissipation associated with highly mobile polymer chains [48]. Importantly, no sharp inflections, plateaus, or discontinuities in *G′*, *G″*, or tan(*δ*) were observed, indicating the absence of premature gelation or uncontrolled crosslinking in the precursor. From a processing perspective, temperatures below the *G′*/*G″* crossover (approximately 43 °C) preserve a predominantly viscous-dominated state, whereas at higher temperatures, the precursor remains highly deformable but exhibits a slightly greater elastic contribution despite continued softening.

Steady-shear rheology was examined through shear-rate sweep measurements to assess flow behavior under processing-relevant conditions (Fig. 3c). The PBIS precursor exhibited strong shear-thinning behavior, with viscosity decreasing substantially as shear rate increased. At low-shear rates, high viscosity reflects entangled chain conformations, whereas at higher shear rates, chain alignment along the flow direction reduces viscous resistance. Shear stress increased smoothly with shear rate, indicating stable flow without yield stress or shear-induced instabilities [49]. This combination of high low-shear viscosity and rapid viscosity reduction under shear is advantageous for fabrication techniques, such as extrusion, molding, or coating [45]. Together, these complementary rheological measurements establish that the uncrosslinked PBIS precursor exhibits entanglement-dominated viscoelasticity, thermal stability, and favorable shear-thinning behavior, providing a well-defined and robust processing window prior to thermal or metal-mediated crosslinking.

#### Thermal Crosslinking Kinetics of Dual-Crosslinked PBIS Elastomers

The evolution of network structure during thermal crosslinking was monitored by isothermal time sweep measurements at 120 °C for PBIS (Batch 7) containing Fe^3+^ ions and the thermal initiator ACVA (Fig. 3d). At the onset of the isothermal curing stage, *G′* and *G″* were of similar magnitude (*G′* ≈ 3000 Pa; *G″* ≈ 2000–3000 Pa), with tan(*δ*) ≈ 0.80, indicating a weakly elastic but highly dissipative coordination-mediated network prior to covalent crosslink formation. As curing progressed over 15,000 s (approximately 4.2 h), *G′* increased steadily to ~ 25000–26000 Pa (approximately eight- to ninefold), while *G″* increased more gradually to ~ 11000 Pa (approximately four-to-fivefold). Correspondingly, tan(*δ*) decreased from ~ 0.80 to ~ 0.44, reflecting a progressive shift toward more elastic-dominated behavior as covalent crosslinks formed and reinforced the coordination-mediated framework. The rate of modulus increase was highest during the first ~ 3000 s (approximately 50 min), after which the growth gradually decelerated but did not fully plateau within the experimental time window. This behavior is consistent with thermally activated radical crosslinking kinetics, where early rapid network formation is followed by reduced reaction rates due to decreased mobility and progressive consumption of reactive double bonds [50]. Importantly, *G′* remained greater than *G″* throughout the measurement, indicating that the system behaved as a solid-like network from the outset and did not undergo a classical sol–gel transition during curing. Instead, thermal treatment densified an already percolated coordination network through formation of permanent covalent crosslinks via ACVA-initiated polymerization of itaconate groups [51]. The preferential increase in *G′* relative to *G″* [as evidenced by the decreasing tan(*δ*)] confirms that covalent crosslinking strengthens the elastic component of the response. The resulting dual-crosslinked architecture combines permanent network integrity with reversible metal–ligand interactions, providing enhanced stiffness while preserving viscoelastic adaptability.

### Physicochemical Stability and Degradation of PBIS Elastomers

#### Metal-Dependent Degradation Behavior

The degradation behavior of M-PBIS elastomers was evaluated using an accelerated alkaline degradation protocol [11]. Disk-shaped M-PBIS (Batch 2) films (6 mm diameter × 1 mm thickness) were incubated in PBS containing NaOH at 37 °C, and degradation was quantified by monitoring mass remaining over time (Fig. 3e,f). Representative optical images (Fig. 3e) revealed metal-dependent morphological change over 72 h, including surface roughening, fragmentation, and discoloration, consistent with progressive polymer network breakdown under alkaline hydrolytic conditions. In contrast, poly(*ε*-caprolactone) (PCL, *M*_*n*_ = 80,000 Da) control samples retained their overall morphology over the same incubation period, indicating substantially greater resistance to base-catalyzed ester hydrolysis. Quantitative analysis revealed a pronounced dependence of degradation rate on metal identity (Fig. 3f). Mg-PBIS exhibited the fastest degradation, with mass remaining decreasing from 88.32% ± 4.89% at 6 h to 55.87% ± 10.17% at 36 h, and further to 11.25% ± 7.35% at 72 h. Fe-PBIS also degraded rapidly, retaining only (22.62% ± 10.06% at 72 h. In contrast, Zn-PBIS and Cu-PBIS showed more gradual mass loss. Zn-PBIS retained 94.25% ± 2.15%, 86.12% ± 5.45%, and 59.37% ± 8.94% of its original mass at 6, 36, and 72 h, respectively. Similarly, Cu-PBIS maintained 94.65% ± 1.51% at 6 h, 82.75% ± 6.52% at 36 h, and 72.93% ± 5.98% at 72 h, indicating substantially higher resistance to alkaline hydrolysis compared to Mg- and Fe-coordinated networks. As a control, PCL exhibited negligible degradation, retaining > 97% mass at all measured time points. These quantitative data indicate that the degradation kinetics of PBIS elastomers are strongly governed by metal–ligand coordination chemistry, with metal ion selection enabling systematic tuning of degradation behavior. It is noted that the accelerated alkaline degradation protocol was employed exclusively as a comparative tool to establish the relative degradation rate ordering among the four M-PBIS formulations under controlled and standardized conditions, rather than to simulate physiological degradation kinetics. The physiologically relevant degradation behavior of the selected Zn-PBIS formulation was subsequently evaluated through subcutaneous implantation in vivo, as described in Sect. 2.9.2.

#### Swelling Characteristics in Physiological Conditions

The swelling behavior of M-PBIS (Batch 2) elastomers was evaluated under physiological conditions by measuring water uptake of disk-shaped films (6 mm diameter × 1 mm thickness) after incubation at 37 °C for 7 and 14 days (Fig. 3g) [52]. Quantitative swelling ratios revealed clear, metal-dependent differences in network stability. At day 7, Zn-PBIS and Cu-PBIS exhibited relatively low swelling ratios of 1.07% ± 0.47% and 1.20% ± 0.32%, respectively, indicating limited water uptake. In contrast, Mg-PBIS showed significantly higher swelling of 3.60% ± 0.91%, while Fe-PBIS exhibited intermediate swelling of 2.13% ± 0.66%. PCL displayed minimal swelling of 0.67% ± 0.30%, consistent with its hydrophobic polyester backbone. Swelling increased for all PBIS formulations between day 7 and day 14, reflecting time-dependent water diffusion and network relaxation. Mg-PBIS and Fe-PBIS showed the largest increases, reaching 5.15% ± 1.32% and 6.36% ± 1.59%, respectively. In comparison, Zn-PBIS and Cu-PBIS exhibited more modest swelling of 2.25% ± 0.62% and 1.64% ± 0.50%, while PCL remained minimally swollen at 0.95% ± 0.30%. The elevated swelling of Mg-PBIS and Fe-PBIS is consistent with their faster degradation behavior, suggesting lower effective crosslink density and greater coordination lability that facilitate water penetration and network expansion. Conversely, the restrained swelling of Zn-PBIS and Cu-PBIS reflects more stable coordination-mediated crosslinks that preserve network integrity. Importantly, all M-PBIS elastomers exhibited swelling ratios below 7%, which falls well within the acceptable range for vascular tissue engineering applications where controlled dimensional stability is critical to maintain lumen patency and mechanical integrity. These modest swelling suggest that M-PBIS elastomers would retain the structural dimensions under physiological conditions without compromising graft function [53].

#### Surface Wettability and Hydrophobicity

Surface wettability of M-PBIS (Batch 2) elastomers was evaluated by static water contact angle measurements to assess surface hydrophobicity (Fig. 3h) [11]. All M-PBIS elastomers exhibited moderate wettability, with contact angles ranging from approximately 62° to 72°, depending on the coordinated metal ion. Mg-PBIS and Fe-PBIS showed the lowest contact angles, of (62.14 ± 4.93)° and (63.82 ± 2.71)°, respectively, indicating relatively higher surface wettability. Zn-PBIS exhibited an intermediate contact angle of (67.22 ± 5.41)°, while Cu-PBIS showed the highest contact angle among the PBIS formulations, at (71.52 ± 2.24)°. In contrast, PCL displayed a substantially higher contact angle of (87.43 ± 1.50)°, consistent with its more hydrophobic polyester surface. The reduced contact angles of PBIS elastomers relative to PCL suggest enhanced surface wettability, which is favorable for protein adsorption and subsequent cell–material interactions [54]. Notably, the relatively narrow range of contact angles across M-PBIS elastomers indicates that metal coordination exerts only a modest influence on surface wettability. This suggests that metal–ligand coordination primarily governs bulk properties such as degradation and swelling, while surface chemistry remains comparatively consistent across different PBIS elastomers.

### Biocompatibility of Zn-PBIS Elastomers

Zn-PBIS was selected for subsequent biological studies based on complementary mechanical and biological considerations, although different formulations were used at different stages of evaluation. Zn-PBIS (Batch 2) was first chosen for in vitro cytotoxicity testing and subcutaneous implantation to assess initial biocompatibility and biodegradation behavior. Among the metal ions evaluated, Mg^2+^ was excluded, because it provided insufficient network reinforcement for load-bearing vascular applications. While Cu^2+^ and Fe^3+^ offered strong mechanical performance, both present potential biological concerns for chronic implantation. Cu^2+^ may be cytotoxic at elevated concentrations [55], whereas Fe^3+^ can promote reactive oxygen species generation through Fenton-type chemistry, potentially inducing oxidative stress in vascular tissue [56]. In contrast, Zn^2+^ provided a balanced mechanical profile together with well-established pro-regenerative roles in vascular biology, including promotion of endothelial cell proliferation, collagen synthesis, and angiogenesis. Zn^2+^ is also known to support endothelial nitric oxide synthase (eNOS) activity, enhance vasoprotection, and modulate macrophage polarization toward less pro-inflammatory phenotypes [57]. Following subcutaneous implantation, however, Zn-PBIS (Batch 2) was found to degrade faster than expected. For improved safety in the vascular setting, the formulation was therefore adjusted to Zn-PBIS (Batch 7) for vascular graft fabrication and subsequent end-to-end interposition grafting in the rat carotid artery model. Together, these considerations motivated the focused evaluation of Zn-coordinated PBIS materials in the biological and vascular graft studies that follow.

#### In Vitro Cytocompatibility and Cell Proliferation

The in vitro cytocompatibility of Zn-PBIS elastomers was evaluated using human umbilical vein endothelial cells (HUVECs) cultured on Zn-PBIS solution-coated glass coverslips. The coatings were prepared following the same coating and curing protocol reported previously for chelation-crosslinked elastomers [11]. PCL-coated coverslips were used as a reference control (Fig. 3i). Live/dead staining at day 3 revealed high cell viability on Zn-PBIS coatings, with HUVECs exhibiting a well-spread morphology and minimal dead cells, comparable to PCL. No evidence of acute cytotoxicity or compromised cell attachment was observed on Zn-PBIS surfaces. Cell growth was further quantified by metabolic activity measurements over 1, 3, and 5 days (Fig. 3i). HUVECs cultured on Zn-PBIS demonstrated a time-dependent increase in metabolic activity, indicating sustained cell growth. At later time points, proliferation on Zn-PBIS was comparable to or slightly higher than that on PCL, suggesting that Zn-PBIS supports endothelial cell growth at least as effectively as the clinically used polyester control. Together, these results demonstrate that Zn-PBIS coatings are cytocompatible and capable of supporting endothelial cell adhesion and proliferation in vitro.

#### In Vivo Evaluation of Biocompatibility and Biodegradation

To evaluate biocompatibility and biodegradation under physiologically relevant conditions, subcutaneous implantation studies were performed in mice (Fig. 3j,k). Zn-PBIS disks (6 mm in diameter × 1 mm in thickness) were implanted subcutaneously and compared with size-matched PCL controls. Implants were monitored for up to 120 days to assess tissue response and material degradation. Macroscopic examination revealed distinct differences in biodegradation behavior between Zn-PBIS and PCL (Fig. 3j). Zn-PBIS implants exhibited progressive degradation over time, with gradual reductions in size and changes in appearance by day 120, while remaining intact and retrievable at all time points, indicating controlled biodegradation without premature fragmentation. In contrast, PCL implants largely retained their original size and morphology throughout the study, consistent with its slow degradation profile [53]. Both materials demonstrated good integration with surrounding tissue, with no visible signs of abscess formation, excessive fibrous encapsulation, or fluid accumulation.

Histological evaluation using hematoxylin and eosin (H&E) staining provided further insight into the tissue–material interface (Fig. 3k). At day 14, both Zn-PBIS and PCL implants were surrounded by a thin fibrous capsule with moderate inflammatory cell infiltration, consistent with an expected early foreign body response. By day 30, Zn-PBIS implants showed evidence of early stage degradation accompanied by increased cellular infiltration at the implant interface, while PCL maintained a relatively stable capsule with minimal changes. At later time points, Zn-PBIS implants exhibited progressive material resorption with increased cell infiltration and a reduction in implant cross-sectional area, while maintaining a thin and organized fibrous capsule, suggesting resolution of inflammation rather than chronic foreign body response. By day 120, substantial degradation of Zn-PBIS was observed. In contrast, PCL implants remained largely intact with a persistent but non-inflammatory fibrous capsule. Overall, neither Zn-PBIS nor PCL elicited severe inflammatory responses, granuloma formation, or tissue necrosis throughout the 120-day implantation period. These results demonstrate that Zn-PBIS elastomers exhibit favorable in vivo biocompatibility while undergoing controlled biodegradation, supporting their potential use in applications requiring temporary structural support and gradual material resorption.

### Fabrication of Architected Zn-PBIS Vascular Grafts

#### Proof-of-Concept 3D Printing of Zn-PBIS via Stereolithography

**Fig. 4:**
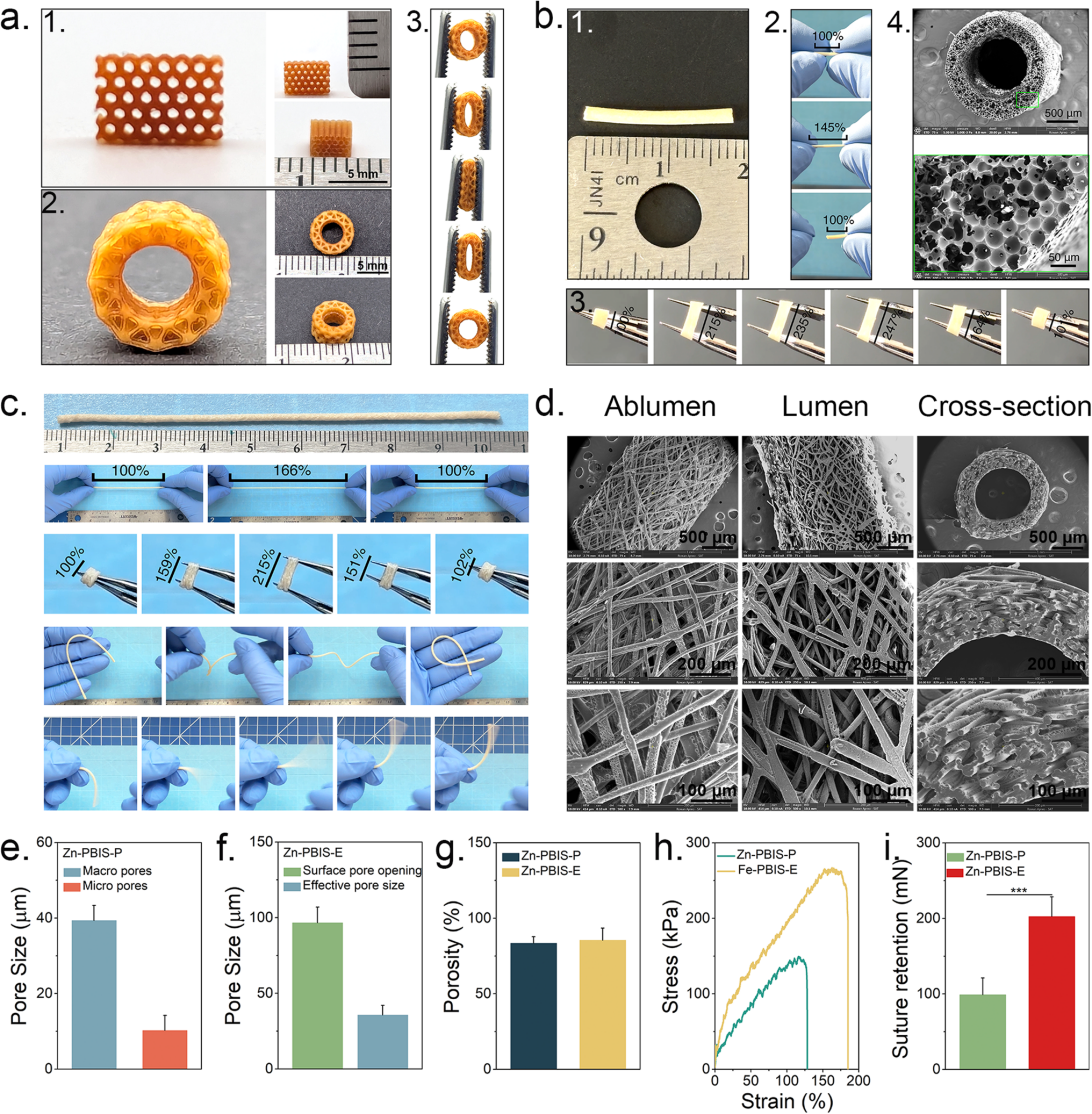
Fabrication, structure, and mechanical performance of Zn-PBIS vascular grafts. **a** Proof-of-concept digital light processing (DLP) 3D-printed Zn-PBIS structures. **b** PMMA-templated porous grafts (Zn-PBIS-P): macroscopic images, deformation under axial and circumferential loading, and representative cross-sectional scanning electron microscopy (SEM) images. **c**, **d** Electrowritten grafts (Zn-PBIS-E): macroscopic views under stretching, radial expansion, torsion, bending, and knotting and SEM images of abluminal surface, luminal surface, and cross-section. **e** Pore size quantification for Zn-PBIS-P. **f** Surface pore opening and effective pore size for Zn-PBIS-E. **g** Bulk porosity comparison. **h** Uniaxial tensile stress–strain curves. **i** Suture retention strength. Data are presented as mean ± SD (*n* = 4). Statistical significance: *** p < 0.001

As a proof-of-concept demonstration of the photocrosslinkable nature and broad processability of the PBIS platform, stereolithography (SLA) 3D printing was briefly explored [58]. This section is not intended to present SLA printing as a viable vascular graft fabrication route, but rather to confirm material compatibility with light-based fabrication approaches. Using this approach, Zn-PBIS structures with lattice patterns and hollow cylindrical geometries were successfully printed (Fig. 4a1–a3), confirming effective photocrosslinking and macroscale shape fidelity. However, SLA-fabricated structures exhibited limited dimensional accuracy at the microscale required for small-diameter vascular grafts, particularly for thin-walled tubular geometries, making this approach unsuitable for the target application in its current form. These resolution limitations provided a clear rationale for pursuing PMMA particle templating and solution electrowriting as the primary fabrication strategies, both of which offer precise control over wall thickness, pore architecture, and fiber organization at the length scales relevant for small-diameter arterial reconstruction, as described in the following sections.

#### PMMA-Templated Porous Zn-PBIS Vascular Grafts

To enable controlled microporosity within vascular graft walls, a PMMA particle-templating strategy was employed, adapted from previously reported methods [12]. A representative porous Zn-PBIS (Batch 7) vascular graft (designated Zn-PBIS-P) is shown in Fig. 4b, exhibiting uniform tubular geometry with an inner diameter of 1 mm and wall thickness of approximately 250 μm. The Zn-PBIS-P grafts displayed good elastic behavior, recovering their original dimensions after uniaxial stretching to 145% strain and circumferential dilation to 247% strain (Fig. 4b2, 4b3), demonstrating robust mechanical resilience. Scanning electron microscopy (SEM) revealed a highly porous architecture throughout the graft wall (Fig. 4b4). Cross-sectional views showed hierarchical macro- and microporosity resulting from PMMA particle removal.

#### Electrowritten PBIS Vascular Grafts

As a fabrication strategy offering precise control over fiber architecture and pore geometry, solution electrowriting was employed to fabricate fibrous Zn-PBIS (Batch 7) vascular grafts (Zn-PBIS-E). This approach was adapted from previously reported solution electrowriting methods for tubular fibrous conduits, which combine electrospinning with direct-write patterning to enable controlled deposition of continuous fibers in predefined architectures [59]. Compared to conventional electrospinning, solution electrowriting provides substantially improved control over fiber placement, spacing, and scaffold geometry. Electrowritten Zn-PBIS grafts achieved the target inner diameter (1 mm) and wall thickness (approximately 200 μm) (Fig. 4c). The grafts exhibited complete elastic recovery following uniaxial stretching to 166% strain and circumferential dilation to 215% strain, and demonstrated excellent flexibility and kink resistance during bending, twisting, and knotting (Fig. 4c), indicating mechanical robustness and suitability for surgical handling and implantation. SEM revealed a highly organized fibrous architecture (Fig. 4d). Both luminal and abluminal surfaces displayed aligned fibers with uniform diameters, while cross-sectional views confirmed a multilayered tubular structure formed by precisely stacked fiber layers.

#### Architecture-Dependent Mechanical Performance of Zn-PBIS Grafts

The architectural and mechanical performance of Zn-PBIS-P and Zn-PBIS-E grafts were systematically compared to assess their suitability for in vivo implantation. Architectural analysis revealed distinct differences arising from the two fabrication approaches. Zn-PBIS-P grafts exhibited a hierarchical porous structure consisting of macropores (39.46 ± 3.98) μm and micropores (10.22 ± 4.01) μm, with an overall porosity of 83.52% ± 4.27% (Fig. 4e, g). In contrast, Zn-PBIS-E grafts displayed larger surface pore openings (96.63 ± 10.31) μm and an effective pore size of (35.64 ± 6.41) μm, defined by the three-dimensional fiber spacing across stacked electrowritten layers, with a slightly higher overall porosity of 85.45% ± 8.06% (Fig. 4f, g). Tensile stress–strain testing revealed elastomeric behavior for both graft types, with pronounced architecture-dependent differences (Fig. 4h). Zn-PBIS-P grafts exhibited a gradual increase in stress, reaching approximately 150 kPa at approximately 130% strain. Zn-PBIS-E grafts reached higher stresses of approximately 280 kPa at approximately 180% strain, reflecting increased stiffness and load-bearing capacity imparted by the aligned fibrous architecture produced through solution electrowriting. The organized fiber reinforcement enables more efficient stress transfer compared to the randomly distributed porous network of PMMA-templated grafts. Suture retention testing further highlighted these architectural effects. Zn-PBIS-E grafts demonstrated significantly higher suture retention forces of (202.58 ± 26.06) mN compared to Zn-PBIS-P grafts at (98.94 ± 22.20) mN (Fig. 4i, ***p < 0.001), consistent with more effective stress distribution within the continuous, aligned fiber network of electrowritten grafts.

### In Vivo Functional Performance of Zn-PBIS Vascular Grafts

#### Gross Appearance and Ultrasound Assessment in a Rat Carotid Artery Interposition Model

**Fig. 5:**
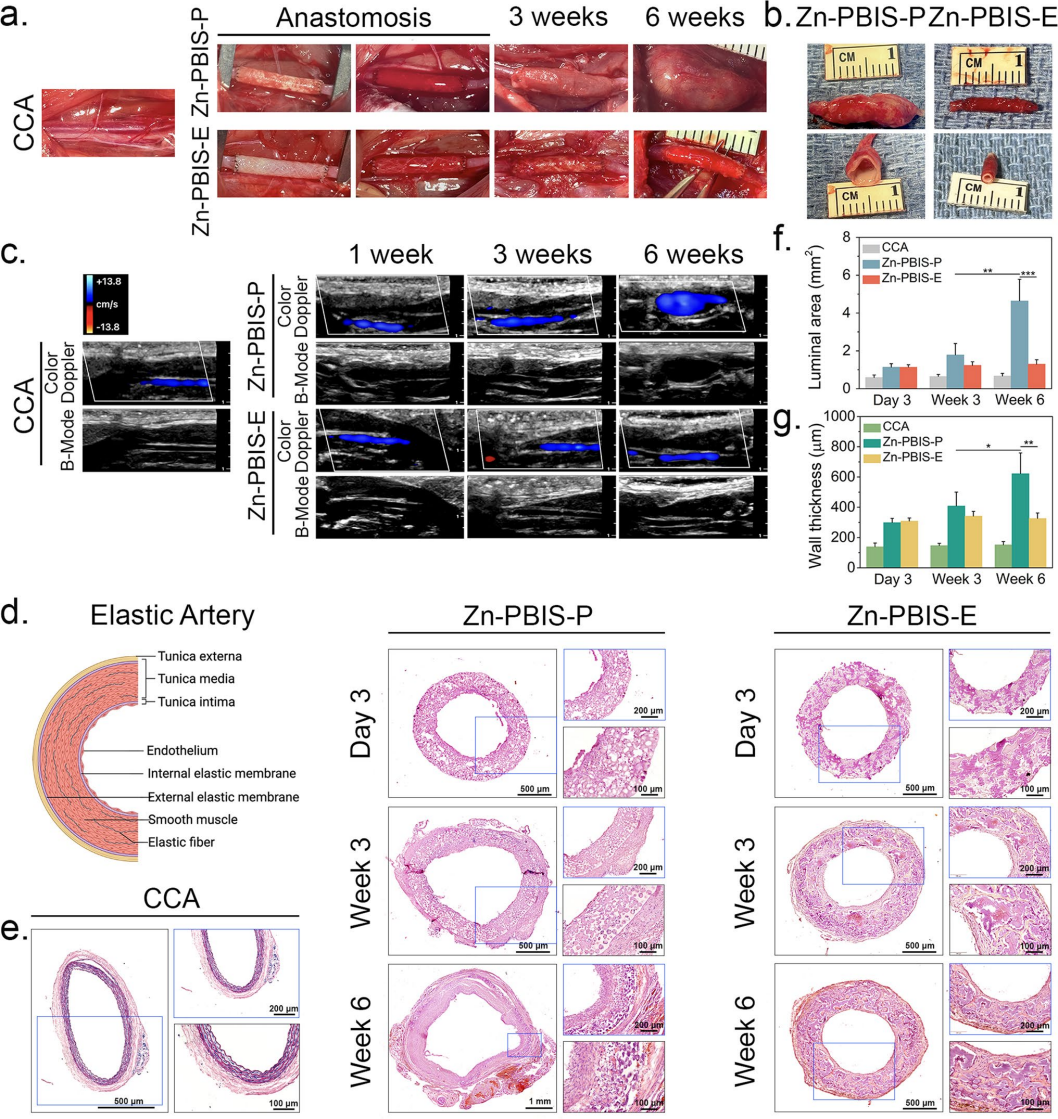
In vivo performance of Zn-PBIS vascular grafts in a rat carotid artery interposition model. **a** Gross images of Zn-PBIS-P and Zn-PBIS-E grafts at anastomosis and at 3 and 6 weeks post-implantation. **b** Cross-sectional gross views of explanted grafts at 6 weeks. **c** Ultrasound images of the native common carotid artery (CCA) and implanted grafts at 1, 3, and 6 weeks. **d** Schematic of native elastic artery wall structure. **e** Representative cross-sectional H&E-stained images of native CCA and explanted grafts at day 3, week 3, and week 6. **f, g** Quantitative luminal area (mm^2^) and wall thickness (μm) from histological cross-sections. Data are presented as mean ± SD (*n* = 4). Statistical significance: * p < 0.05, ** p < 0.01, *** p < 0.001

To assess the in vivo functional performance of the Zn-PBIS vascular grafts under physiological arterial conditions, Zn-PBIS-P and Zn-PBIS-E grafts were implanted as interposition conduits in a rat common carotid artery (CCA) model. This established small-diameter arterial model subjects grafts to pulsatile blood flow and arterial pressure, providing a stringent platform to evaluate graft suturability, patency, hemodynamic stability, and architecture-dependent vascular remodeling in vivo [60]. Representative intraoperative and longitudinal gross images are shown in Fig. 5a. During implantation, both Zn-PBIS-P and Zn-PBIS-E grafts demonstrated excellent surgical handling, tolerating suturing without tearing and enabling secure end-to-end anastomoses. Immediately after reperfusion, both grafts restored blood flow with visible pulsatile expansion, indicating successful hemodynamic integration with the host vasculature. By week 3, Zn-PBIS-P grafts began to exhibit visible luminal dilation while remaining patent, whereas Zn-PBIS-E grafts preserved a vessel caliber comparable to the native CCA. By week 6, Zn-PBIS-P grafts showed pronounced dilation (Fig. 5a,b), although without rupture. In contrast, Zn-PBIS-E grafts maintained a stable tubular geometry with no gross evidence of aneurysmal expansion. Explanted grafts at week 6 (Fig. 5b) confirmed these observations, with enlarged lumens observed in Zn-PBIS-P grafts and well-defined, stable lumens retained in Zn-PBIS-E grafts.

Longitudinal noninvasive ultrasound imaging was used to further assess graft patency, blood flow, and qualitative changes in luminal opening over the 6-week implantation period (Fig. 5c) [12]. Patency was maintained in 14/18 Zn-PBIS-P grafts and 17/22 Zn-PBIS-E grafts throughout the study, with intraluminal blood flow detected at all imaging time points. At 1-week post-implantation, color Doppler imaging revealed organized flow through both graft types, indicating unobstructed blood passage. Over time, B-mode imaging revealed progressive luminal expansion in Zn-PBIS-P grafts, while Zn-PBIS-E grafts preserved a more uniform luminal profile with stable flow patterns. These gross and ultrasound observations demonstrate that while both graft architectures support surgical implantation and sustained patency, scaffold architecture strongly influences in vivo dimensional stability, motivating quantitative morphometric analysis of lumen and wall evolution in the following section.

#### Histological and Quantitative Morphometric Analysis

Histological and morphometric analyses were performed to quantify grafts performance following carotid interposition (Fig. 5d–g). Native elastic artery structure is shown as a reference (Fig. 5e), exhibiting a well-organized multilayered wall with a thin neointima, circumferential smooth muscle layers, and preserved elastic lamellae [61]. At day 3 post-implantation, both Zn-PBIS-P and Zn-PBIS-E grafts maintained patent lumens with intact scaffold architecture (Fig. 5e, top row). The graft walls were readily distinguishable from native tissue, with Zn-PBIS-P displaying a porous morphology and Zn-PBIS-E exhibiting a fibrous, layered structure. Early cellular infiltration was observed primarily at the graft periphery. By week 3, both graft types demonstrated substantial cellular infiltration throughout the scaffold thickness, accompanied by increased cellular density and early extracellular matrix deposition (Fig. 5e, middle row). Zn-PBIS-P grafts exhibited early luminal expansion, reflected by an increase in luminal area relative to native vessels (Fig. 5f). In contrast, Zn-PBIS-E grafts preserved a more uniform luminal opening while supporting controlled wall thickening. Neointimal formation was evident in both grafts. However, Zn-PBIS-E grafts showed a more continuous and circumferentially organized neointima, whereas Zn-PBIS-P grafts exhibited a less uniform neointimal layer associated with progressive dilation.

At week 6, divergent remodeling outcomes were observed (Fig. 5e, bottom row). Zn-PBIS-P grafts displayed severe aneurysmal dilation with marked loss of circular geometry and dramatic luminal enlargement, consistent with progressive mechanical failure. Quantitatively, Zn-PBIS-P grafts showed a pronounced increase in luminal area and excessive wall thickening (Fig. 5f,g), indicating loss of dimensional control during remodeling. Histologically, the porous scaffold structure was no longer continuous; instead, fragmented remnants of the original PMMA-templated matrix were visible as discontinuous regions within a highly cellularized but disorganized tissue wall. In contrast, Zn-PBIS-E grafts maintained luminal patency and dimensional stability at week 6, with luminal areas comparable to earlier time points and significantly smaller than those of Zn-PBIS-P grafts (Fig. 5f). Wall thickness increased modestly but remained well controlled (Fig. 5g), consistent with constructive tissue remodeling rather than pathological dilation. Histological sections revealed an intact, circumferentially organized graft wall with integrated fibrous scaffold elements supporting uniform tissue formation. Together, these quantitative and histological findings demonstrate that while both graft architectures support early cellular infiltration and tissue formation, only the electrowritten Zn-PBIS-E grafts maintain mechanical and geometric stability during the critical remodeling phase. The random porous architecture of Zn-PBIS-P grafts permits rapid tissue ingrowth but fails to preserve radial integrity under arterial loading as scaffold degradation progresses, leading to aneurysmal dilation.

### Vascular Remodeling and Mechanistic Interpretation

#### Cellular Infiltration and Phenotype Organization

**Fig. 6:**
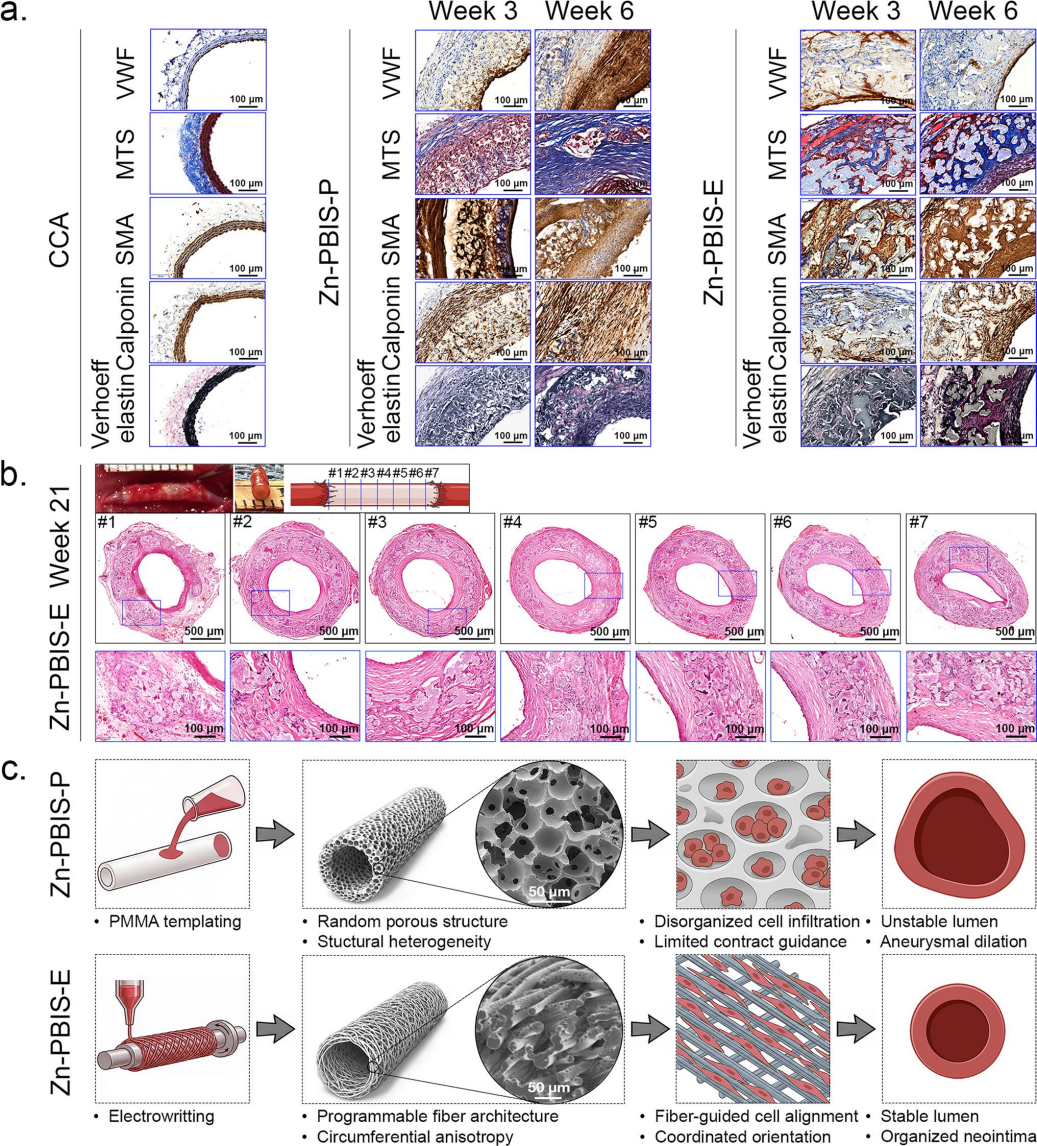
In vivo remodeling and tissue integration of Zn-PBIS vascular grafts. **a** Histological and immunohistochemical staining of CCA, Zn-PBIS-P, and Zn-PBIS-E at weeks 3 and 6 after implantation, including von Willebrand factor (vWF), Masson’s trichrome staining (MTS), α-smooth muscle actin (α-SMA), calponin, and Verhoeff elastin staining. **b** Serial cross-sectional H&E-stained images of Zn-PBIS-E grafts at 21 weeks across seven positions along the graft length. **c** Schematic comparing architecture-dependent remodeling outcomes in Zn-PBIS-P and Zn-PBIS-E grafts

To elucidate the cellular mechanisms underlying architecture-dependent vascular remodeling, immunohistochemical analyses were performed at 3 and 6 weeks post-implantation, with native CCA serving as a reference. At week 3 (Fig. 6a, S18), von Willebrand factor (vWF) staining indicated the early establishment of luminal endothelialization in both Zn-PBIS-P and Zn-PBIS-E grafts. Masson’s Trichrome staining (MTS) revealed pronounced architecture-dependent differences in extracellular matrix organization. Zn-PBIS-P grafts exhibited heterogeneous and spatially discontinuous collagen deposition distributed throughout the porous scaffold, reflecting uncoordinated matrix formation. In contrast, Zn-PBIS-E grafts showed more continuous and circumferentially oriented collagen deposition aligned with the fibrous scaffold architecture, indicating guided matrix organization under arterial loading. α-Smooth muscle actin (α-SMA) and calponin staining demonstrated early smooth muscle cell infiltration in both graft types, with cells in Zn-PBIS-E grafts exhibiting preferential alignment along the electrowritten fibers. Elastin deposition remained minimal in both groups at week 3, consistent with an early remodeling phase preceding elastic fiber maturation.

By week 6 (Fig. 6a, S19), immunohistochemical analyses revealed pronounced architecture-dependent differences in endothelial integrity, matrix organization, and medial maturation. vWF staining demonstrated a continuous, thin, and luminally confined vWF-positive layer in Zn-PBIS-E grafts, consistent with stable endothelialization and preserved luminal architecture. In contrast, Zn-PBIS-P grafts exhibited intense and spatially expanded vWF staining extending into a markedly thickened neointimal region. This broadened vWF signal coincided with luminal irregularity and structural disruption, indicating injury-associated endothelial activation and thrombo-inflammatory remodeling rather than formation of a stable endothelial monolayer [62]. MTS staining of Zn-PBIS-P grafts showed collagen deposition with an overall circumferential tendency; however, the collagen network was loosely organized, spatially heterogeneous, and interrupted by large porous domains, resulting in a structurally discontinuous vessel wall with regional thickening. In contrast, Zn-PBIS-E grafts displayed a continuous and densely organized circumferential collagen architecture integrated with aligned cellular layers.

Smooth muscle organization also diverged markedly between graft architectures. Zn-PBIS-P grafts exhibited intense but spatially disorganized α-SMA expression throughout a markedly thickened vessel wall, accompanied by heterogeneous and diffusely distributed calponin staining, consistent with reactive myofibroblast-like remodeling. In contrast, Zn-PBIS-E grafts showed α-SMA^+^ cells with pronounced circumferential alignment and more spatially confined calponin expression, forming organized cellular layers indicative of guided smooth muscle organization toward a developing tunica media [63]. Elastin deposition, further underscored these differences. Zn-PBIS-P grafts exhibited fragmented and spatially discontinuous elastin confined to isolated regions, consistent with dysregulated elastogenesis during aneurysmal remodeling [64]. In contrast, Zn-PBIS-E grafts showed more uniformly distributed elastin with preferential circumferential alignment following the electrowritten fiber architecture. Although elastin content remained lower than native carotid arteries, this organized distribution suggests early scaffold-guided elastogenesis accompanying constructive arterial remodeling. Collectively, these findings demonstrate that scaffold microarchitecture governs not only cellular infiltration but also the spatial organization and phenotypic maturation of endothelial cells, smooth muscle cells, and extracellular matrix components, ultimately dictating divergent remodeling trajectories under physiological arterial loading.

#### Long-Term Structural Stability and Spatial Remodeling of Zn-PBIS-E Grafts

To assess long-term durability and spatial remodeling, Zn-PBIS-E grafts were harvested at 21 weeks (Supplemental Movie 1) and analyzed by H&E staining across seven serial cross-sections spanning the proximal anastomosis to the distal end (#1–#7, Fig. 6b). Gross inspection demonstrated complete tissue integration and preserved tubular geometry without evidence of aneurysmal dilation, stenosis, or external constriction. The graft appeared well vascularized and structurally continuous. Serial H&E sections revealed uniform luminal patency across all seven positions. Luminal geometry remained circular to mildly elliptical without focal narrowing or dilation, indicating sustained mechanical stability under chronic arterial loading. Wall thickness was relatively consistent along the graft length, and no segment exhibited structural discontinuity or collapse. Importantly, no region demonstrated excessive neointimal overgrowth or focal degeneration, indicating spatially balanced remodeling.

Low-magnification views demonstrated a well-organized, multilayered vessel wall resembling native arterial architecture. The graft exhibited dense cellularity with discernible inner and outer zones, consistent with progressive structural maturation. Residual scaffold fibers were still detectable within the wall, reflecting sustained but controlled degradation kinetics. Importantly, these remnants were well integrated within the neotissue and did not result in wall thinning, structural discontinuity, or luminal compromise, indicating coordinated scaffold resorption and tissue formation. Higher-magnification analysis revealed elongated nuclei within the medial region exhibiting preferential circumferential alignment, consistent with organized smooth muscle-like remodeling. The outer wall displayed more heterogeneous cellular orientation, compatible with adventitial maturation. Abundant extracellular matrix deposition formed a cohesive and mechanically integrated vessel wall. Importantly, circumferential tissue organization was preserved across all sampled positions, indicating that scaffold-guided alignment had transitioned into a stable, self-maintained structural architecture. Collectively, these findings demonstrate sustained patency, dimensional stability, and spatially uniform remodeling at 21 weeks, without evidence of aneurysmal degeneration or segmental failure.

#### Architecture-Driven Remodeling Mechanisms

Integration of the structural, morphometric, immunohistochemical, and long-term data reveals two fundamentally distinct remodeling pathways governed by scaffold architecture (Fig. 6c). Although both Zn-PBIS-P and Zn-PBIS-E grafts permitted cellular infiltration and matrix deposition, their long-term outcomes diverged due to differences in architectural guidance and mechanical anisotropy. The PMMA-templated Zn-PBIS-P grafts possessed a random porous structure that enabled rapid cellular invasion but provided limited directional cues and no coherent circumferential reinforcement. Infiltrating cells distributed throughout the pore network without coordinated alignment, leading to heterogeneous smooth muscle organization and spatially discontinuous extracellular matrix deposition. While collagen deposition exhibited a partial circumferential tendency, it remained mechanically fragmented by pore-induced structural interruptions. As scaffold degradation progressed, the absence of a circumferential load-bearing framework reduced resistance to radial arterial stress, predisposing the graft to progressive dilation and aneurysmal remodeling [65]. Thus, although the porous architecture supported infiltration, it failed to instruct organized tissue assembly capable of sustaining long-term mechanical stability.

In contrast, the electrowritten Zn-PBIS-E grafts were fabricated with a circumferentially-biased helically wound fibrous architecture that recapitulated the anisotropic organization of native arteries. This geometry provided both directional contact guidance and circumferential load-bearing reinforcement. Infiltrating cells adhered to and migrated along aligned fibers, adopting preferential circumferential orientation that guided collagen and elastin deposition along the same axis. The resulting coordinated cell–matrix organization established a mechanically coherent medial-like layer capable of resisting radial expansion. As scaffold degradation advanced, organized neotissue progressively replaced the synthetic framework while preserving circumferential structural integrity, thereby maintaining stable lumen geometry and long-term durability under physiological loading. Together, these results establish scaffold microarchitecture as a primary regulator of arterial remodeling stability. Rather than serving as a passive template for cell infiltration, the electrowritten architecture actively encodes mechanical anisotropy and spatial guidance that couples cellular alignment with load-bearing matrix assembly [66]. This geometry-dependent mechanobiological coupling governs the transition from scaffold-supported structure to tissue-dominated stability, defining a critical design principle for durable small-diameter vascular graft engineering.

In addition to scaffold architecture, the choice of metal ion coordinating the PBIS network may provide a complementary bioactive contribution to vascular remodeling. Beyond its structural role as a dynamic coordination crosslinker, Zn^2+^ released during scaffold degradation may also contribute to the constructive vascular remodeling observed in Zn-PBIS grafts. Zn^2+^ is an essential trace element involved in endothelial cell function, extracellular matrix remodeling, and inflammatory regulation. Gradual Zn^2+^ release from the degrading Zn-PBIS network may, therefore, support endothelialization, smooth muscle cell organization, collagen deposition, and modulation of the local inflammatory microenvironment [67]. In the present study, both Zn-PBIS-P and Zn-PBIS-E grafts were fabricated from the same Zn-coordinated polymer chemistry; therefore, their distinct remodeling outcomes are attributed primarily to differences in scaffold architecture. Nevertheless, Zn^2+^ release may provide a shared bioactive contribution that supports vascular remodeling in both graft types. Future studies will systematically decouple the effects of metal ion identity, ion release kinetics, and graft architecture by comparing Zn-, Mg-, Cu-, and Fe-coordinated PBIS grafts under matched architectural conditions.

## Conclusion

This study establishes M-PBIS as a programmable metallo-elastomer platform for small-diameter vascular graft applications, combining reversible metal–ligand coordination with covalent crosslinking to achieve broad tunability in stiffness (0.06–3.2 MPa), extensibility (53%–491%), toughness (66–1339 kJ m^−3^), and degradation kinetics. Systematic tuning of diacid composition, bipyridine ligand density, itaconate content, and metal ion identity demonstrated that dual-crosslinked networks consistently outperformed single-crosslinked networks in mechanical stability, autonomous self-recovery, and sustained load-bearing capacity. Beyond material design, scaffold microarchitecture proved decisive in determining vascular remodeling outcomes. While PMMA-templated porous grafts permitted cellular infiltration but resulted in disorganized matrix deposition and aneurysmal dilation by week 6, electrowritten grafts with biomimetic circumferentially-biased fiber alignment actively guided smooth muscle organization, coordinated extracellular matrix assembly, and maintained stable lumen geometry through 21 weeks of arterial implantation, demonstrating that scaffold architecture functions as an active regulator of vascular morphogenesis.

Despite these promising results, several important limitations remain. Validation in large-animal models under clinically relevant hemodynamic conditions remains necessary. Complete scaffold resorption and functional assessments, including compliance matching, vasoactivity, and endothelial function testing, have yet to be performed. While the present study demonstrates controlled biodegradation in subcutaneous and vascular implantation models, a direct quantitative correlation between scaffold degradation kinetics and neotissue formation rate was not established. Future work will focus on time-resolved quantification of scaffold remnant content and extracellular matrix maturation at matched implantation time points, to provide a more mechanistic understanding of how degradation and tissue regeneration are temporally coupled in the M-PBIS system. Future work should, therefore, focus on large-animal validation, extended implantation studies, scalable manufacturing development, and time-resolved degradation-remodeling correlation studies.

## Experimental Section

PBIS polymers were synthesized by solvent-assisted step-growth polyesterification of diethyl[2,2′-bipyridine]-5,5′-dicarboxylate, succinic acid, sebacic acid, itaconic acid, and 1,3-propanediol using titanium(IV) isopropoxide as catalyst under nitrogen, followed by bulk polycondensation under reduced pressure and purification by precipitation. Polymer composition and physicochemical properties were characterized by quantitative ^1^H NMR, advanced polymer chromatography (APC), differential scanning calorimetry (DSC), thermogravimetric analysis (TGA), Fourier-transform infrared spectroscopy (FTIR), and UV–Vis spectroscopy.

Metal-coordinated PBIS networks were prepared by mixing PBIS with metal chloride salts (Fe^3+^, Zn^2+^, Cu^2+^, or Mg^2+^) at defined metal-to-ligand ratios. Dual-crosslinked networks were generated by introducing either thermal covalent crosslinking with 4,4′-azobis(4-cyanovaleric acid) (ACVA) or photocrosslinking with trimethylolpropane tris(3-mercaptopropionate) (TMPMP) and lithium phenyl-2,4,6-trimethylbenzoylphosphinate (LAP). Mechanical performance was evaluated by uniaxial tensile, cyclic, creep, and rheological testing, and self-healing was assessed using a cut-and-rejoin protocol followed by tensile retesting.

Zn-PBIS vascular grafts were fabricated by digital light processing (DLP) 3D printing, PMMA microsphere-templated porogen leaching, and near-field solution electrowriting. DLP printing used a photocrosslinkable Zn-PBIS resin, whereas porous and electrowritten grafts were prepared from Zn-PBIS using thermal curing and subsequent washing/porogen removal steps. Graft morphology, pore size, porosity, tensile properties, and suture retention were evaluated by scanning electron microscopy and mechanical testing under wet conditions.

In vitro cytocompatibility was evaluated using HUVECs cultured on Zn-PBIS substrates. In vivo biocompatibility and biodegradability were assessed by subcutaneous implantation of Zn-PBIS disks in BALB/cJ mice. Vascular graft performance was evaluated in a rat carotid artery interposition model using ethylene oxide-sterilized, heparin-treated Zn-PBIS grafts, followed by ultrasonographic monitoring and histological/immunohistochemical analysis at predefined time points. All animal procedures were approved by the Rowan University Cooper Medical School IACUC and conducted in accordance with NIH guidelines.

Detailed procedures are provided in the Supporting Information.

## Supplementary Information

Below is the link to the electronic supplementary material.

Supplementary file1 (MP4 5332 KB)

Supplementary file2 (DOCX 36663 KB)

## Data Availability

The data supporting the findings of this study are available from the corresponding author upon reasonable request.
