# Supplementary material for: A Dual-Crosslinked Metallo-elastomer Platform for Architecture-Directed Vascular Remodeling
Source: Adv Fiber Mater. 2026 Jul 4;8(5):2160–84. doi: 10.1007/s42765-026-00744-9 (PMC13468867; doi:10.1007/s42765-026-00744-9)
Supplement: Supplementary file 1 [file 42765_2026_744_MOESM2_ESM.docx]

**A Dual-Crosslinked Metallo-Elastomer Platform for Architecture-Directed Vascular Remodeling**

Narangerel Gantumur^a,†^, Shuhao Jiao^a,†^, Xiaochu Ding^b,c^, Simon Van Herck^d^, Isabella Frangiosa^a^, Emily Kopchick^a^, Caitlin Maureen Purdy^e^, Ty Walker^a^, Aarati Kharal^a^, Ying Grace Chen^a^*

a. Department of Biomedical Engineering, Henry M. Rowan College of Engineering, Rowan University, 201 Mullica Hill Road, Glassboro, NJ 08028, USA
b. Health Research Institute, Michigan Technological University, H-STEM 238, 1400 Townsend Drive, Houghton, MI 49931, USA
c. Department of Chemistry, Michigan Technological University, 1400 Townsend Drive, Houghton, MI 49931, USA
d. Department of Instructive Biomaterials Engineering, MERLN Institute for Technology-Inspired Regenerative Medicine, Maastricht University, Universiteitssingel 40, 6229 ER Maastricht, the Netherlands
e. Center for Research and Education in Advanced Transportation Engineering Systems (CREATES) of the Henry M. Rowan College of Engineering, Rowan University, 201 Mullica Hill Road, Glassboro, NJ 08028, USA

^†^ These authors contributed equally to this work.

*Corresponding author:

Ying Grace Chen, Ph.D., Email: [chenyin@rowan.edu](mailto:chenyin@rowan.edu)

**Experimental section**

**1 Chemicals and Reagents**

Diethyl[2,2′-bipyridine]-5,5′-dicarboxylate (Ethyl-DP, 97%, Catalog #77010-434) was purchased from VWR (Radnor, PA, USA). Succinic acid (SU), sebacic acid (SA), itaconic acid (IA), 1,3-propanediol (PDO), titanium(IV) isopropoxide (Ti(OiPr)₄), and 4-methoxyphenol were obtained from Sigma-Aldrich (St. Louis, MO, USA). Anhydrous iron(III) chloride (FeCl₃), zinc chloride (ZnCl₂), copper(II) chloride (CuCl₂), and magnesium chloride (MgCl₂) were purchased from Sigma-Aldrich and used as metal coordination agents. 4,4′-Azobis(4-cyanovaleric acid) (ACVA), trimethylolpropane tris(3-mercaptopropionate) (TMPMP), and lithium phenyl-2,4,6-trimethylbenzoylphosphinate (LAP) were obtained from Sigma-Aldrich and used as thermal or photo-initiated crosslinking agents. Chloroform, methanol, absolute ethanol, 1,1,1,3,3,3-hexafluoro-2-propanol (HFIP), N,N-dimethylformamide (DMF), 1,4 dioxane, tetrahydrofuran (THF), and phosphate-buffered saline (PBS) were purchased from Sigma-Aldrich and used as received. Deuterated chloroform (chloroform-d, CDCl₃, 99.95% atom D) was purchased from Sigma-Aldrich and used as the solvent for ^1^H NMR spectroscopy. Poly(methyl methacrylate) (PMMA) microspheres (38–45 μm diameter) were obtained from Cospheric (Santa Barbara, CA, USA) and used as sacrificial porogens for templated graft fabrication. Poly(ε-caprolactone) (PCL, *M_n_* =80,000 Da) was purchased from Sigma-Aldrich and used as a reference control material. All chemicals were used as received unless otherwise noted.

**2 PBIS Polymer Synthesis**

**2.1 Solvent-assisted step-growth polyesterification**

PBIS polymers were synthesized via solvent-assisted step-growth polyesterification of diethyl[2,2′-bipyridine]-5,5′-dicarboxylate (Ethyl-DP), succinic acid (SU), sebacic acid (SA), itaconic acid (IA), and 1,3-propanediol (PDO) (Fig. 1a). Diethyl 2,2′-bipyridine-5,5′-dicarboxylate was used instead of the corresponding dicarboxylic acid due to its significantly lower melting point and good solubility in dioxane, enabling homogeneous solution-assisted polymerization [1]. The free acid (>360 °C melting point) is poorly soluble in dioxane and unsuitable for the selected reaction conditions. Polymerizations were conducted in a 100 mL three-neck round-bottom flask equipped with mechanical stirring under a dry nitrogen atmosphere. Reactions were performed on a total diacid scale of 0.01 mol. As a representative formulation, Batch 2 (10% DP, 40% SU, 40% SA, 10% IA; Figure S1) was prepared using 0.001 mol Ethyl-DP (0.3003 g), 0.004 mol succinic acid (0.4724 g), 0.004 mol sebacic acid (0.809 g), 0.001 mol itaconic acid (0.1301 g), and 0.01 mol 1,3-propanediol (0.7609 g, 722.2 μL). 4-Methoxyphenol (5 mg, 1 mol% to itaconic acid) was added as a radical inhibitor to preserve the vinyl functionality of itaconate during thermal processing [2]. Titanium (IV) isopropoxide (20 mg, 5 mol% to Ethyl-DP) was used as the esterification catalyst. Anhydrous 1,4-dioxane (3 mL) was added to facilitate monomer dissolution.

Prior to heating, the reaction system was purged with nitrogen for 2 h to remove oxygen and moisture. The reaction flask was then immersed in a silicone oil bath set to 140 °C with continuous stirring. During the first 4 h, the condenser was positioned to maintain continuous reflux of 1,4-dioxane. Under these conditions, solvent vapor condensed and returned to the reaction flask, preserving a homogeneous solution-phase reaction environment. Initial esterification and transesterification occurred during this reflux stage, and oligomer formation was evidenced by a gradual increase in viscosity. After 4 h of reflux, the condenser orientation was adjusted to permit controlled distillation. At this stage, dioxane together with condensation byproducts, including water and ethanol, was allowed to distill into the receiving flask. As solvent content decreased, the system progressively transitioned from solution-phase conditions to bulk melt polymerization. The reaction was maintained under nitrogen at the oil bath temperature to promote further molecular weight growth. When sufficient viscosity was achieved (~ 16 h), the reaction mixture was subjected to polycondensation under reduced pressure (~10⁻² Torr) using a two-stage rotary vane vacuum pump (Edwards RV3). Residual solvent and low-molecular-weight species were removed under vacuum to further drive condensation and increase molecular weight. The reaction was terminated when the viscosity increased to the point that stirring was significantly impeded (12-18 h). After cooling to room temperature, the crude polymer was dissolved in DMF and precipitated into excess cold absolute ethanol to remove unreacted monomers. The precipitated polymer was washed repeatedly with ethanol and dried under vacuum at 40 °C for 24 h. Dried polymers were stored at −20 °C until further use.

**2.2 Control of compositional variables**

The PBIS platform was designed to enable systematic modulation of backbone structure and network functionality through controlled variation of three compositional parameters: (i) diacid ratio (SA/SU), (ii) bipyridine ligand content (DP), and (iii) itaconate content (IA). In all formulations, the total diacid equivalents were maintained stoichiometric with 1,3-propanediol to preserve step-growth polymerization balance. The theoretical monomer feed compositions for all formulations (Batches l–7) are summarized in Fig. S1. Backbone crystallinity and chain flexibility were tuned by varying the molar ratio of sebacic acid (SA) to succinic acid (SU). SA contains a longer C10 aliphatic segment that promotes chain regularity and semicrystalline packing, whereas SU introduces shorter C4 spacers that disrupt crystalline ordering and increase segmental mobility [3]. By adjusting the SA/SU ratio, formulations spanning SA-only (PBIS__SA_), SU-only (PBIS__SU_), and equimolar compositions (PBIS__SA/SU=1_) were prepared to investigate the influence of diacid structure on thermal and mechanical behavior.

Metal coordination site density was regulated by varying the molar fraction of DP incorporated into the polymer backbone. Low (PBIS__DP-L_, batch 4), medium (PBIS__DP-M_, batch 2), and high (PBIS__DP-H_, batch 5) ligand-density formulations were synthesized to control the number of bipyridine moieties available for subsequent metal-ligand coordination. Increasing DP content proportionally increased the density of potential coordination junctions, thereby providing a means to modulate network reinforcement through metal-mediated crosslinking [4].

Covalent crosslinkable site density was controlled by adjusting the molar fraction of itaconic acid (IA) within the total diacid feed. As IA is a diacid comonomer, increases in IA content were compensated by proportional adjustments in SA and/or SU content to maintain constant overall diacid stoichiometry. Itaconate units introduce pendant vinyl (C=C) functionalities capable of radical-mediated crosslinking [5]. Low (PBIS__IA-L_, batch 6), medium (PBIS__IA-M_, batch 2), and high (PBIS__IA-H_, batch 7) IA formulations were prepared to vary the density of vinyl groups available for thermal or photo-initiated network formation. Within this subset, DP content was held constant to isolate the effect of IA incorporation on covalent network formation.

**2.3 Determination of polymer composition by quantitative ^1^H NMR**

Polymer composition was determined by ^1^H nuclear magnetic resonance (^1^H NMR) spectroscopy [6]. PBIS polymer (10 mg) was dissolved in deuterated chloroform (CDCl_3_, 99.95% atom D) and transferred into 5 mm NMR tubes. Spectra were recorded at 400 MHz on a Varian Mercury 400 MHz NMR spectrometer (Varian Inc., USA) at room temperature. Chemical shifts were referenced to residual CHCl_3_ ($\delta=7.26$ ppm). For each polymer batch, three independently prepared samples were analyzed by ^1^H NMR ($n=3$). Representative spectra for Batches 1–7 are shown in Fig. 1b and Figs. S2–S7, with peak assignments corresponding to the individual monomer units. Characteristic signals from the DP units were identified from aromatic proton resonances (h, i, and j) in the 8.2 l–9.4 ppm region. The vinyl protons of itaconate units (g) were observed at approximately 5.6 l–6.4 ppm. Aliphatic protons from sebacic acid (SA), succinic acid (SU), and 1,3-propanediol (PDO) were assigned in the 1.2 l–4.3 ppm region.

The DP content (DP%) and itaconate content (IA%) were quantified from diagnostic proton signals in the ^1^H NMR spectra (Figs. S2–S8). Bipyridine ligand content was determined from the combined integration of aromatic protons h, i, and j (6 H per DP unit), while itaconate content was calculated from the combined integration of vinyl protons g (2 H per itaconate unit). All integral area ratios were normalized to the O-CH_2_ protons of 1,3-propanediol (peak d, 4 H per repeat unit), which served as the internal reference for all polymer batches.

Here, $y$represents the molar fraction of DP, $w$represents the molar fraction of IA, $x$represents the molar fraction of SU, and $z$represents the molar fraction of SA in the polymer backbone, with $w+x+y+z=1$. The molar fractions of DP and IA were calculated according to:

$$y= \frac{4}{6} \left( \frac{A_{h+i+j}}{A_{d}} \right) \left( 1 \right)$$

$$w= 2 \left( \frac{A_{g+g}}{A_{d}} \right) \left( 2 \right)$$

where $A_{h+i+j}$, $A_{g}$, and $A_{d}$correspond to the integral areas of the bipyridine aromatic protons, the combined itaconate vinyl protons, and the PDO O-CH_2_ protons, respectively.

Because the succinate methylene signal (peak f, 4 H per SU unit) partially overlaps with the methylene signal of the itaconate unit (2 H per IA unit), the succinate integral was corrected as:

$$\begin{matrix} & A_{f,\mathrm{SU}}=A_{f}-2w & & \text{(3)} \end{matrix}$$

where $A_{f}$is the measured integral area of peak f, and $w$is the molar fraction of IA determined from Equation (2). The SU and SA molar fractions were then calculated from the corrected succinate signal and the sebacate-specific mid-chain methylene signal (peak c, 8 H per SA unit) according to:

$$\begin{matrix} & x=\frac{A_{f,\mathrm{SU}}/4}{A_{f,\mathrm{SU}}/4+A_{c}/8}\text{ }(1-y-w) & & \text{(4)} \end{matrix}$$

$$\begin{matrix} & z=\frac{A_{c}/8}{A_{f,\mathrm{SU}}/4+A_{c}/8}\text{ }(1-y-w) & & \text{(5)} \end{matrix}$$

where $A_{c}$is the integral area of the sebacate mid-chain methylene protons (peak c), and $1-y-w$represents the combined molar fraction of SA and SU, i.e., the remaining fraction after excluding DP and IA contributions. Calculated molar percentages for all batches are summarized in the insets in Fig. S2-S8.

**3 Structural and Physicochemical Characterization of PBIS**

**3.1 Fourier-transform infrared (FTIR) spectroscopy**

Fourier-transform infrared (FTIR) spectra of pristine PBIS and Fe^3^⁺-coordinated PBIS (batch 2) samples were collected using a Thermo Scientific™ Nicolet™ iN10 FTIR spectrometer (Thermo Fisher Scientific) operated in attenuated total reflectance (ATR) mode. Fe-coordinated PBIS samples were prepared as described in Section 4.1. Spectra were recorded over a wavenumber range of 600–3600 cm^-1^ with a spectral resolution of 4 cm^-1^. Background spectra were collected prior to each sample and automatically subtracted. All measurements were conducted at room temperature. The measurements were repeated triplicate (n=3).

**3.2 UV-Vis spectroscopic determination of Fe^3+^:PBIS coordination ratio**

The Fe^3+^:PBIS coordination ratio was evaluated by UV-Vis absorption spectroscopy using a SpectraMax M3 microplate reader (Fig. 1d). PBIS (Batch 2) was dissolved in chloroform to obtain a homogeneous solution (1 mg mL^-1^). A methanolic solution of FeCl₃ was added incrementally to the PBIS polymer (batch 2) solution to achieve Fe^3+^: bipyridine molar ratios ranging from 1:6, 1:5, 1:4, 1:3, 1:2, 1:1, to 2:1, calculated based on the experimentally determined DP% (Section 2.3). After each addition, the solution was mixed and diluted 2000× and allowed to equilibrate for approximately 5 min prior to spectral acquisition. Control spectra were collected for (i) PBIS solution without Fe^3+^, and (ii) FeCl₃ solution in methanol at corresponding concentrations. UV-Vis spectra were recorded at room temperature between 220 and 500 nm using quartz cuvettes with 1 cm optical path length. The measurements were repeated triplicate (n=3).

**3.3 Advanced polymer chromatography (APC)**

The number-average (*M_n_*) and weight-average (*M_w_*) molecular weights, as well as dispersity (PDI = *M_w_*/*M_n_*), of PBIS polymers were determined using a Waters ACQUITY™ Advanced Polymer Chromatography (APC) system (Waters Corporation, USA) equipped with a refractive index (RI) detector. Tetrahydrofuran (THF, Optima™ grade) was used as the eluent. Molecular weight calibration was performed using narrow-dispersity polystyrene standards spanning the relevant molecular weight range. Samples were dissolved in THF at 5 mg mL⁻¹, filtered through a 0.45 μm PTFE syringe filter prior to injection, and analyzed at a column temperature of 35 °C. Molecular weight values are reported as mean ± standard deviation from three independent measurements (*n* = 3).

**3.4 Differential scanning calorimetry (DSC)**

Thermal properties of PBIS polymers were characterized using a TA Instruments Model 2920 Modulated Differential Scanning Calorimeter (TA Instruments, USA). Samples (5.00–10.00 mg) were hermetically sealed in aluminum Tzero pans with matching Tzero lids (TA Instruments) and analyzed under a nitrogen atmosphere. The thermal program consisted of equilibration at –90 °C followed by a 5 min isothermal hold, heating to 250 °C at 10 °C min⁻¹, and holding isothermally for 5 min at 250 °C. Samples were subsequently cooled to –90 °C at 5 °C min⁻¹. A second heating scan from –90 °C to 250 °C at 10 °C min⁻¹ was performed, and thermal transition temperatures were determined from the second heating cycle unless otherwise specified. All measurements were performed in triplicate (*n* = 3).

**3.5 Thermogravimetric analysis (TGA)**

Thermogravimetric analysis was performed using a TA Instruments Model 2950 Hi-Resolution Thermogravimetric Analyzer (TA Instruments, USA). Polymer samples (3.5-10.5 mg) were placed in open platinum pans and analyzed under a nitrogen atmosphere to prevent oxidative degradation. Nitrogen was purged at a constant flow rate (100.0 ml/min). Samples were heated from room temperature to 500 °C at a linear heating rate of 10 °C min⁻¹. The temperature corresponding to 10% weight loss (*T_10%_*), the maximum degradation temperature (*T_max_*), and the residual mass at 495 °C were recorded. All measurements were conducted in triplicate (*n* = 3).

**4 Metal Coordination, Covalent Crosslinking, and Dual Network Formation**

**4.1 Preparation of metal-PBIS coordination networks**

Metal-PBIS coordination networks (M-PBIS) were prepared by blending the PBIS polymer solutions with metal salt solutions followed by solvent casting and controlled drying [4]. PBIS polymers were first dissolved in chloroform at 20–30 wt% under magnetic stirring overnight at room temperature to obtain homogeneous polymer solutions. Metal salts including ZnCl₂, FeCl₃, CuCl₂, and MgCl₂·6H₂O were dissolved in alcohol solvents to prepare concentrated stock solutions. ZnCl₂ and FeCl₃ were respectively dissolved in methanol at 200 mg mL⁻¹. CuCl₂ was dissolved in ethanol at 200 mg mL⁻¹, and MgCl₂·6H₂O was dissolved in methanol at 100 mg mL⁻¹. All metal solutions were freshly prepared to minimize hydrolysis and were vortexed until complete dissolution for use.

The required amount of metal salt was determined based on the bipyridine ligand molar fraction (DP%) obtained from ^1^H NMR analysis. For a given PBIS mass (*m*_PBIS_), the number of moles of repeat units was calculated using the composition-weighted average molar mass of the repeat unit (*M*_repeat_):

$$n_{repeat}=\frac{m_{PBIS}}{M_{repeat}}$$

The number of moles of bipyridine ligands was then determined as:

$$n_{DP}=n_{repeat}\times(DP mol\%)$$

Metal ion quantities were adjusted according to the expected coordination stoichiometry. For divalent metal ions (Zn^2+^, Cu^2+^, Mg^2+^), a 1:2 metal-to-ligand molar ratio was employed, corresponding to:

$$n_{M^{2+}}=\frac{1}{2}n_{DP}$$

For Fe^3+^ systems, a 1:3 metal-to-ligand molar ratio was used:

$$n_{Fe^{3+}}=\frac{1}{3}n_{DP}$$

consistent with octahedral tris(bipyridine) coordination [4].

The calculated volume of metal salt solution was added dropwise to the PBIS solution under vigorous vortex mixing (1–2 min) to ensure uniform dispersion and rapid coordination. The mixed solution remained optically homogeneous without visible precipitation, indicating successful coordination within the polymer matrix. The resulting Metal-PBIS solution was cast into silicone molds to produce films with typical thicknesses of 0.5–1 mm. Solvent evaporation was first carried out in a chemical fume hood under ambient conditions for 12–24 h. The films were then transferred to a vacuum oven (Thermo Scientific™ Lindberg/Blue M) connected to a two-stage rotary vane pump (Edwards RV3) and dried at room temperature under reduced pressure (~10⁻² Torr) for an additional 12 h to ensure complete removal of residual solvent. Films were then heated to 120 °C for 24 h in a vacuum oven to facilitate metal-ligand coordination [4]. After curing, samples were allowed to cool down to room temperature, removed from the molds, and stored in a desiccator until further use.

**4.2 Thermal crosslinking via ACVA**

Thermal covalent crosslinking was carried out via radical polymerization of pendant itaconate (vinyl) groups using 4,4′-azobis(4-cyanovaleric acid) (ACVA) as a thermal initiator [7]. ACVA was freshly dissolved in methanol at a concentration of 68 mg mL⁻¹ immediately prior to use. The amount of ACVA was calculated as 5 mol% relative to the molar amount of itaconate (IA) units in the polymer based on the ^1^H NMR analysis. The calculated volume of ACVA solution was added to the PBIS solution under stirring, and the mixture was vortexed for 1–2 min to ensure homogeneous distribution. The resulting solution was cast into silicone molds as described above. Solvent evaporation was first conducted in a chemical fume hood under ambient conditions for 12–24 h, followed by thermal curing at 120 °C for 24 h in a convection oven to initiate radical crosslinking. After curing, samples were allowed to cool down to room temperature, removed from the molds, and stored in a desiccator until further use.

**4.3 Photo-crosslinking via TMPMP and LAP**

Photo-induced covalent crosslinking of PBIS was performed via thiol-ene click reaction between pendant itaconate double bonds and thiol groups in trimethylolpropane tris(3-mercaptopropionate) (TMPMP). The amount of TMPMP was calculated at 1.2 equivalents relative to the molar amount of itaconate (IA) units, as determined from experimentally measured IA mol% by ^1^H NMR analysis. This slight excess of thiol functionality was employed to promote efficient reactions with the carbon-carbon double bonds and ensure complete network formation. TMPMP was added directly to the PBIS solution (30–40 wt% in chloroform) under vortex mixing for 1–2 min to ensure homogeneous distribution. Lithium phenyl-2,4,6-trimethylbenzoylphosphinate (LAP) was used as the photoinitiator at 5 mol% relative to IA units and was pre-dissolved in methanol (100 mg mL⁻¹) immediately prior to use. The calculated volume of LAP solution was added to the polymer mixture, followed by brief vortex mixing to achieve uniform dispersion. The resulting precursor solution was cast into silicone molds and irradiated using a 365 nm UV LED lamp (Everbeam 365 nm, 100 W) at a fixed working distance of 10 cm for 20 min per side. After UV exposure, the crosslinked elastomeric films were removed from molds and dried under vacuum at room temperature to remove residual solvent prior to characterization.

**4.4 Dual-crosslinked network fabrication**

Dual-crosslinked PBIS networks were fabricated by sequential formation of metal-ligand coordination interactions followed by covalent crosslinking through either thermal or photo-initiated radical polymerization. In all cases, metal coordination was introduced prior to covalent curing to establish supramolecular crosslinks between bipyridine ligands and metal ions. Briefly, PBIS was dissolved in chloroform (20–30 wt%) to obtain a homogeneous solution. A calculated volume of metal salt solution (ZnCl₂, FeCl₃, CuCl₂, or MgCl₂·6H₂O), prepared in methanol or ethanol, was added to achieve the desired metal-to-bipyridine molar ratio based on experimentally determined DP% values. The mixture was vortexed for 1–2 min to ensure uniform metal distribution and allowed to equilibrate to promote coordination complex formation.

For metal and thermal dual-crosslinked networks, ACVA (5 mol% relative to IA units) was added to the metal-coordinated PBIS solution as described in Section 4.2. The mixture was cast into silicone molds, solvent was allowed to evaporate under ambient conditions, and thermal curing was carried out at 120 °C for 24 h to induce radical polymerization of itaconate double bonds. For metal and photo dual-crosslinked networks, TMPMP (1.2 equiv relative to IA units) and LAP (5 mol% relative to IA units) were added sequentially after metal coordination as described in Section 4.3. The precursor solution was cast into molds and irradiated under 365 nm UV light (10 cm working distance, 20 min per side) to generate covalent thiol-ene crosslinks. Fully cured dual-crosslinked elastomers were stored in a desiccator prior to characterization.

**5 Mechanical Testing**

Elastomeric PBIS films for mechanical evaluation were prepared as described in Sections 4.2–4.4. After curing, rectangular specimens were cut from uniform regions of the films using a precision blade to ensure consistent geometry. Film thickness was measured using a digital micrometer and ranged from approximately 0.8 to 1.2 mm. The gauge length for each specimen was manually measured prior to testing. Mechanical testing was performed using a Shimadzu Mechanical Tester (EZ-SX model) equipped with Trapezium X software and a 500 N load cell. Uniaxial tensile tests were conducted at room temperature at a constant crosshead speed of 20 mm min⁻¹. Stress was calculated based on the initial cross-sectional area of each specimen. Young’s modulus was determined from the linear region of the stress-strain curve at low strain (<20% strain) using linear regression. The ultimate tensile stress (UTS) and strain at fracture were obtained directly from the stress-strain curves. Toughness was calculated by integration of the area under the stress-strain curve up to failure. For each formulation, four independent specimens (*n* = 4) were tested.

Cyclic tensile tests were performed at room temperature using the same instrument at a crosshead speed of 30 mm min^-1^. Specimens were subjected to 200 consecutive loading-unloading cycles to a maximum strain of 20% [4]. Hysteresis was quantified by calculating the area enclosed within each loading-unloading loop, and the evolution of peak stress and energy dissipation over cycling was analyzed to evaluate mechanical resilience and viscoelastic response. Four independent specimens (*n* = 4) were tested for each condition.

Creep behavior was evaluated under constant uniaxial tensile stress using the same mechanical tester. Rectangular specimens prepared as described above were mounted with a gauge length of 5–10 mm. Tests were conducted at 37 °C to simulate physiological conditions. Constant stresses of 0.1, 0.5, and 1 MPa were applied, and strain was continuously recorded over a 24 h period. The crosshead speed was set to 1 mm min^-1^ during initial loading to reach the target stress, after which stress was maintained constant throughout the creep duration. The time-dependent strain response was analyzed to evaluate primary creep (instantaneous elastic deformation), secondary creep (steady-state deformation), and long-term viscoelastic flow behavior. Three independent specimens were tested for each stress level (*n* = 3).

**6 Self-Healing Assessment**

Macroscopic self-healing behavior of M-PBIS elastomer films was evaluated using a complete cut-and-rejoin protocol. Dual-crosslinked M-PBIS films were cut entirely into two separate pieces using a razor blade to generate clean fracture interfaces. The freshly cut surfaces were immediately brought back into contact with slight manual overlap to ensure continuous interfacial contact and eliminate air gaps. The overlapped interface was gently pressed and aligned to restore geometric continuity of the specimen. A 200 g weight was applied normal to the interface to promote polymer chain reorganization and reformation of M-bipyridine coordination bonds. Healing was conducted at 37 °C for 1 h. After the healing period, the applied weight was removed, and the specimens were carefully handled to assess macroscopic integrity. Healed samples were then subjected to uniaxial tensile testing under the same conditions described in Section 5. Four independent specimens (*n* = 4) were tested for both pristine and self-healed conditions. Self-healing efficiency was evaluated by comparing mechanical parameters (UTS, elongation at break, modulus, and toughness) of healed samples relative to pristine samples.

**7 Rheological Characterization**

Rheological measurements were performed using a Kinexus Ultra^+^ rotational rheometer (Malvern Panalytical, UK) equipped with rSpace 2.0 software for instrument control and data acquisition [8]. A parallel-plate configuration with 8 mm diameter stainless steel upper and lower plates was used for all experiments. Prior to each measurement, both plates were preheated to the desired test temperature and zeroed using the instrument control software. PBIS precursor samples were loaded onto the lower plate, and the upper plate was lowered to establish a fixed gap of 0.4 mm. Excess material was carefully trimmed to avoid edge effects. The environmental temperature control hood was then closed, and samples were allowed to equilibrate for 2–3 min after the temperature stabilized within ± 0.2 °C before testing commenced. Frequency sweep measurements of the uncrosslinked PBIS precursor were conducted at 37 °C under 2% oscillatory shear strain, which was confirmed to lie within the linear viscoelastic region. The angular frequency was increased linearly over 50 points from 0.1592 to 15.92 Hz (equivalent to 1–100 rad s^-1^). Storage modulus (*G′*), loss modulus (*G′′*), and complex viscosity (*η**) were recorded as functions of frequency. Amplitude sweep experiments were also performed at 37 °C to determine the linear viscoelastic regime. Shear strain was increased logarithmically from 0.1% to 10% at a constant frequency of 1.592 Hz, using 10 strain increments.

Steady shear measurements were conducted at 25 °C to evaluate flow behavior. Samples were allowed to equilibrate for 180 s under zero shear prior to testing. The shear rate was increased from 1 to 100 s^-1^ using five data points per decade, and viscosity and shear stress were recorded continuously. Temperature sweep measurements were performed from 25 to 120 °C at a heating rate of 3 °C min^-1^ under oscillatory shear at 1.592 Hz and 1.0% strain. Storage modulus, loss modulus, and tan(*δ*) were monitored as functions of temperature to assess thermal softening behavior of the precursor prior to crosslinking. For crosslinking kinetics measurements of Fe-PBIS systems, samples were loaded at 25 °C and heated to 120 °C. After 1 min of thermal equilibration, oscillatory time sweep measurements were performed at 1.0% strain and 1.592 Hz for up to 4 h under continuous data acquisition. The evolution of *G′*, *G′′*, and tan(*δ*) was recorded to monitor network formation during thermal curing.

**8 Degradation, Swelling, and Surface Wettability**

Accelerated degradation, equilibrium swelling, and surface wettability of M-PBIS films were systematically evaluated to assess hydrolytic stability, network integrity, and interfacial properties. Accelerated alkaline degradation was performed under basic conditions to evaluate hydrolytic susceptibility of ester linkages within the polymer backbone [4]. Disk-shaped M-PBIS (batch 2) samples (6 mm diameter × 1 mm thickness) were dried to constant weight and their initial dry mass (m₀) was recorded using a microbalance (Mettler Toledo, XSR105). Each specimen was immersed in 5 mL phosphate-buffered saline (PBS) containing 60 mM NaOH (pH = 12.63) and incubated at 37 °C on a rotating shaker to ensure uniform exposure. At predetermined time points (6, 36, and 72 h), samples were removed, rinsed thoroughly five times with deionized water to eliminate residual salts and base, frozen, and lyophilized for 24 h. The dried mass (*m_t_*) was then measured. The percentage of mass remaining was calculated as: Mass remaining (%) = (*m_t_*/ *m₀*) × 100. Three independent samples (*n* = 3) were analyzed for each condition, and results are reported as mean ± standard deviation.

For equilibrium swelling behavior, the initial dry mass (m₀) of each specimen was recorded prior to immersion. After incubation in deionized water at 37 °C for the designated time points (7 and 14 days), samples were removed, gently blotted to remove excess surface water, and immediately weighed to obtain the swollen mass (m_s_). The equilibrium swelling ratio (Q) was calculated as: Q = (*m_s_* − *m₀*)/ *m₀* [9]. Measurements were performed in triplicate (*n* = 4), and averaged values are reported.

Static water contact angle measurements were performed on cured M-PBIS coatings prepared on glass substrates [10]. Briefly, PBIS (Batch 2) was dissolved in chloroform (1% w/v) to form a homogeneous precursor solution, and ACVA and the desired metal salt (Fe^3+^, Zn^2+^, Cu^2+^, or Mg^2+^) were added as described in the network preparation procedure. 200 μL of each resulting M-PBIS precursor was drop-cast onto one clean glass microscope slide to form a thin, continuous coating layer, respectively. Coated slides were dried to remove solvent and subsequently thermally cured to form crosslinked M-PBIS networks. After curing, samples were rinsed thoroughly with deionized water to remove residual salts or unbound species and dried under vacuum at room temperature prior to wettability testing. PCL (*M_n_* =80,000 Da) coating was prepared as a control by dissolving PCL in chloroform (1% w/v) and directly casting the solution onto glass slides, followed by solvent evaporation under ambient conditions. Sessile-drop contact angle measurements were conducted at room temperature using an optical tensiometer (Attension Theta, Biolin Scientific). A droplet of deionized water (50 μL) was placed on the coated surface, and images were captured immediately after deposition. Contact angles were determined using the instrument software by fitting the droplet profile (Young-Laplace method). Measurements were performed at multiple locations on each coated slide (4 spots per sample), and values were averaged.

**9 *In vitro* cytocompatibility study**

The *in vitro* cytotoxicity assay was performed on the Zn-PBIS (batch 2) coating using Human umbilical vein endothelial cells (HUVECs, C2519A, Lonza) according to the manufacturer’s instructions [11]. The HUVECs (passage 5) were sub-cultured using endothelial cell growth medium that contained 2% FBS and VEGF (EGM-2 BulletKit, CC-3156 & CC-4147, Lonza). The cells were harvested using conventional trypsin/EDTA after reaching confluence and re-suspended in EGM-2 to prepare a cell suspension solution of 5 × 10^5^ cells per mL for cell seeding. 1% w/v of Zn-PBIS in THF was prepared and 20 μL of the solution was evenly spread on each of the coverslips (12 mm diameter). 20 μL of 1% w/v acetone solution of PCL (*M_n_* =80,000 Da) was coated on each of the coverslips to prepare the controls. The Zn-PBIS and PCL coatings with a thickness of approximately 200 nm were formed on the coverslips after the coverslips were air-dried for 24 hours. The Zn-PBIS coatings were further cured in a vacuum oven at 30 mTorr and 120 ℃ for 12 hours. The coated coverslips were placed into 24-well cell culture plates with the coatings orientated upward and then sterilized by UV radiation for 30 min. Each coverslip was soaked in ethanol for 2 h to remove any unreacted monomers, Zn^2+^ or residual solvents. The ethanol was then replaced by PBS and then EGM-2 medium. 20 μL medium which contained 5 × 10^3^ HUVECs cells was added dropwise onto the coverslips and the cells were allowed to attach for 3 hours before 1 mL of EGM-2 medium was added to each well of the cell culture plates. The cells were incubated at 37°C with 5% CO_2_. The medium exchange was carried out every 48 hours. A Vybrant^®^ MTT Cell Proliferation Assay Kit (Invitrogen, CA) was used to measure the cell metabolic activity of the HUVECs after incubated for 1, 3 and 5 days. The absorbance was recorded using a SpectraMax M3 microplate reader. Live/dead assay was performed after cells incubated for 5 days using a LIVE/DEAD^®^ Viability/Cytotoxicity Kit (Invitrogen, CA). The fluorescence microscopy images were recorded using a Nikon AX confocal microscope (Nikon Instruments lnc., NY) to assess cell viability, with live cells ﬂuorescing green and dead cells appearing red. All experiments were performed in triplicate.

**10 *In vivo* biocompatibility and biodegradability study**

All procedures involving live animals were approved by the Rowan University Cooper Medical School Institutional Animal Care and Use Committee (IACUC) and conducted in accordance with institutional guidelines. Ethylene oxide-sterilized Zn-PBIS (Batch 2) and PCL (*M_n_* ≈ 80,000 Da) discs (6 mm diameter × 1 mm thickness) were implanted subcutaneously in eight BALB/cJ mice (Jackson Laboratory) aged 8–9 weeks. Under deep isoflurane-O₂ general anesthesia, small dorsal incisions were made and subcutaneous pockets were created by blunt dissection. Sterile samples were inserted into the pockets, and the incisions were closed using standard surgical procedures [4].

At predetermined time points (14, 30, 60, and 120 days post-implantation), animals were euthanized, and tissue blocks (approximately 15 × 15 mm) containing the implants and surrounding host tissue were harvested en bloc. Explanted tissues were fixed in 4% paraformaldehyde for 1.5 h at room temperature, followed by cryoprotection in 30% sucrose solution for 48 h [12]. Samples were then embedded in Cryomatrix™ embedding resin (Mercedes Scientific) and frozen. Serial cross-sections (8 µm thickness) were collected from the central region of each implant. Sections were stained with hematoxylin and eosin (H&E) using reagents obtained from Electron Microscopy Sciences. Stained sections were imaged using a PreciPoint M8 digital slide scanner at 4×, 10×, and 20× objective magnifications. H&E-stained sections were evaluated for inflammatory cell infiltration, fibrous capsule formation, collagen deposition, tissue integration, and any evidence of adverse host response.

**11 Fabrication of Zn-PBIS vascular Grafts**

**11.1 3D printing of Zn-PBIS vascular grafts**

Photo-crosslinkable Zn-PBIS (Batch 7) prepolymer was used to fabricate elastomeric scaffolds via digital light processing (DLP) 3D printing [13]. The prepolymer (0.8 g) was dissolved in a mixed solvent system consisting of toluene, hexafluoroisopropanol (HFIP), and chloroform (5:5:1 v/v, total 3.3 mL) in a glass vial and vortexed to dissolve at room temperature for 12-24 h. Trimethylolpropane tris(3-mercaptopropionate) (TMPMP, 213 mg, 0.53 mmol) was used as the multifunctional thiol crosslinker. LAP (Lithium phenyl-2,4,6-trimethylbenzoylphosphinate) (24 mg, 0.08 mmol) was added as the photoinitiator [8]. The crosslinker and initiator were combined with the Zn-PBIS prepolymer solution and vortexed for 5 min. The resulting resin was transferred to the duravat of a B9 Series 530 DLP 3D printer (B9Creations, LLC, USA). Printing was performed at a wavelength of 405 nm with a layer thickness of 30μm. The initial foundation layers were exposed at 600 mJ cm⁻², followed by 300 mJ cm⁻² for subsequent layers. Lattice-patterned scaffolds and hollow cylindrical graft geometries were successfully fabricated. After printing, the constructions were carefully removed from the build platform and subjected to a sequential solvent washing protocol to remove residual monomers, initiator, and solvent. Scaffolds were immersed in acetone (4 hours), acetone: DCM (1:1, overnight), acetone (4 hours) followed by graded acetone: ethanol mixtures (4:1, 2:1,1:2, 1:4 v/v; 2 h each). Finally, samples were rinsed in deionized water for 48 h, with water refreshed every 24 h to ensure thorough solvent extraction. Following washing, scaffolds were frozen at −80 °C overnight and subsequently lyophilized for 48 h to preserve structural integrity and internal porosity.

**11.2 PMMA-templated porous Zn-PBIS vascular grafts**

Porous Zn-PBIS (Batch 7) vascular grafts were fabricated via a porogen leaching method using PMMA microspheres as sacrificial templates [14]. A custom cylindrical glass mold (inner diameter = 2.0 mm, length ≈ 15 mm) equipped with a central stainless-steel mandrel (outer diameter = 1.0 mm) was used to define the outer wall and luminal dimensions of the grafts. The 1.0 mm mandrel diameter was selected to approximate the lumen size of the rat carotid artery for subsequent in vivo studies. PMMA microspheres (38–45 μm diameter) were introduced into the mold and packed around the central mandrel. To achieve uniform and compact bead packing, the bead-filled mold was subjected to sonication for approximately 30 min. The packed PMMA beads were then thermally treated at 175 °C for 24 h to partially fuse adjacent microspheres, forming a mechanically stable, interconnected porous template. The fused PMMA template was carefully removed from the mold prior to polymer infiltration. PBIS precursor solution was prepared by dissolving the polymer at a concentration of ~300 mg mL⁻¹ in a 2:1 mixture of ethanol: 1,4 dioxane solution, followed by addition of ZnCl₂ and ACVA at the predetermined ligand-to-metal molar ratio. The Zn-PBIS solution was then added dropwise onto the PMMA template to allow infiltration throughout the template.

After complete infiltration, the polymer-loaded templates were thermally cured at 120°C for 24 h for thermal crosslinking. Following curing, Zn-PBIS/PMMA composite tubes were subjected to solvent extraction to remove the PMMA porogens. Samples were immersed in dichloromethane for up to 7 days, with solvent refreshed every 8–12 h to ensure complete dissolution of PMMA beads. Subsequent sequential washing in acetone, ethanol, and deionized water (24 h each, with periodic solvent replacement) was performed to remove residual solvent and byproducts. The resulting porous Zn-PBIS grafts were frozen at −80 °C and lyophilized for 48 h to preserve pore architecture. Dried grafts were stored in a desiccator at room temperature prior to further characterization or sterilization.

**11.3 Electrowritten Zn-PBIS vascular grafts**

Electrowritten fibrous Zn-PBIS vascular grafts were fabricated using a custom-built near-field solution electrowriting system. Preliminary attempts to directly electrowrite pure Zn-PBIS were unsuccessful due to the short fiber flight distance (< 1 cm) and the intrinsically soft elastomeric nature of PBIS, which resulted in poor jet stability and collapse of deposited fibers on the rotating mandrel. To enhance print fidelity and fiber stability, poly(vinyl alcohol) (PVA, *M_w_* ≈ 74,800) was incorporated as a sacrificial carrier polymer [15]. PBIS (batch 7) and PVA were blended at a mass ratio of 80:20 and dissolved in 1,1,1,3,3,3-hexafluoro-2-propanol (HFIP) to obtain a total polymer concentration of 12% (w/v). Zinc chloride (ZnCl₂) and 4,4′-azobis(4-cyanovaleric acid) (ACVA) were added to the solution prior to electrowriting to enable coordination crosslinking and subsequent thermal covalent crosslinking. The precursor solution was vortex stirred until homogeneous before loading into a syringe fitted with a 27 G blunt needle. Electrowriting was performed at a flow rate of 3.0 μL min⁻¹ under an applied voltage of 3.94 kV. A 1 mm diameter stainless-steel mandrel served as the rotating collector and was driven at 200 rpm using an IKA Eurostar 20 Digital Overhead Stirrer. The translational stage was programmed at 1000 mm min⁻¹ to control fiber spacing and scaffold architecture. Relative humidity was maintained at approximately 15% to promote stable jet formation and minimize solvent condensation. The mandrel was coated with a thin layer of hyaluronic acid to facilitate uniform fiber deposition and subsequent tube removal. Following deposition, the fibrous grafts were thermally crosslinked at 120 °C under vacuum for 24 h for thermal-crosslinking. After curing, scaffolds were immersed in deionized water at 37 °C for eight consecutive 9 h washing cycles to remove the PVA carrier polymer [15]. The water was refreshed between cycles to ensure complete extraction. The resulting electrowritten Zn-PBIS grafts were frozen and lyophilized, and stored in a desiccator prior to further characterization.

**11.4 SEM characterization, porosity analysis, and mechanical testing of vascular grafts**

The morphology of Zn-PBIS vascular grafts was characterized using a FEI Thermo Scientific Apreo S LoVac scanning electron microscope. Grafts were cryo-fractured in liquid nitrogen to obtain clean cross-sections, mounted on aluminum stubs with conductive carbon tape, and sputter-coated with a thin gold layer (~5–10 nm). Surface and cross-sectional images were acquired at multiple magnifications to evaluate pore architecture and fiber organization. Pore dimensions were quantified using ImageJ (NIH). For PMMA-templated porous grafts (Zn-PBIS-P), macro-pores (bead-templated primary pores) and micro-pores (secondary voids within pore walls) were measured from four randomly selected SEM images per graft (500×), with at least 20 pores analyzed per sample. For electrowritten grafts (Zn-PBIS-E), surface pore openings and effective pore sizes were determined by measuring the inter-fiber spacing and the largest inscribed circle within interconnected fiber junctions (≥20 measurements per graft, *n* = 4).

Bulk porosity was determined using a geometric mass–volume method [16]. The dry mass of each graft (m₀) was measured using a microbalance. The total geometric volume (V_total_) was calculated from measured graft dimensions (inner diameter, outer diameter, and length) assuming a cylindrical geometry. The solid polymer volume (V_solid_) was calculated from the dry mass and polymer density (ρ_polymer_): V_solid_ = m₀/ ρ_polymer_. Porosity (%) was calculated as: Porosity = (1 – V_solid_/V_total_) × 100. Measurements were performed in triplicate (*n* = 3).

Mechanical properties of the vascular grafts were evaluated using a Shimadzu EZ-SX mechanical tester. Graft dimensions were measured using a digital caliper. Prior to testing, all samples were soaked in DPBS. A uniaxial force was applied to the grafts until failure occurred. The strain and stress of each sample were recorded to generate a stress-strain curve. The testing process was conducted within a short time frame to ensure the samples remained wet. Four independent samples were tested for each group (*n* = 4). The suture retention force of the grafts was assessed using the same mechanical tester. All test samples were 10 mm in length. A single 9-0 Nylon suture (ARO Surgical) was passed through the specimen, positioned 2 mm from the top edge [14]. The upper clamp of the testing machine held the suture, while the lower clamp secured the nonsutured end of the test sample. The suture was pulled at a constant rate of 20 mm min^⁻1^. All grafts were tested under wet conditions at room temperature. The suture retention force was defined as the maximum force recorded before the suture became dislodged from each sample. For each group, four different samples (*n* = 4) were tested.

**12 *In vivo* performances of Vascular Grafts**

**12.1 Vascular grafts implantation**

All animal procedures were approved by the Institutional Animal Care and Use Committee (IACUC) of the Rowan University Cooper Medical School and were conducted in accordance with the National Institutes of Health (NIH) Guide for the Care and Use of Laboratory Animals. Prior to implantation, vascular grafts were sterilized using ethylene oxide (Andersen Products) and subsequently immersed in sterile heparin solution (180 U mL⁻¹) overnight to enhance hemocompatibility before *in vivo* implantation [14]. A total of 40 male and female SAS Sprague Dawley rats (Charles River Laboratories, Boston, MA), aged 8–12 weeks and weighing 300–450 g, were used for left common carotid artery interposition grafting. The contralateral (right) common carotid artery in each animal served as the unoperated control. Of the total animals, 18 received Zn-PBIS-P grafts, 22 received Zn-PBIS-E grafts. Rats were anesthetized with isoflurane delivered in oxygen (induction at 3.5% isoflurane in 1 L min⁻¹ O₂, followed by maintenance at 1.5%). A midline cervical incision was made to expose the left common carotid artery. Blood flow was temporarily occluded using double microvascular clamps (Fine Science Tools). An approximately 8 mm segment of the native carotid artery was excised, and the vascular graft was interposed between the proximal and distal arterial stumps using end-to-end anastomosis with simple interrupted 10-0 polyamide monofilament sutures (ARO Surgical, Newport Beach, CA). Upon completion of the anastomoses, the clamps were removed to restore blood flow, and patency was visually confirmed. The muscle and skin were closed using 3-0 MONOCRYL absorbable sutures (Y344H, Ethicon). No anticoagulant or antiplatelet therapy was administered postoperatively. Analgesia was provided via a single subcutaneous injection of sustained-release buprenorphine (Buprenorphine SR, 1.0–1.2 mg kg⁻¹), providing up to 72 hours of postoperative pain control.

**12.2 Patency monitoring by ultrasonography**

Graft patency, luminal diameter, and blood flow were longitudinally monitored at 1, 3, 6, and 21 weeks post-implantation using a Butterfly iQ3 Ultrasound System (Butterfly Network, Inc., USA). The contralateral common carotid artery (CCA) served as an internal control. Rats were anesthetized with 1–2% isoflurane delivered in 1 L min⁻¹ oxygen and positioned supine on a temperature-controlled platform. Hair over the cervical region was shaved, and ultrasound gel was applied to ensure acoustic coupling. Transcutaneous ultrasound imaging was performed along the longitudinal axis of the implanted graft and native carotid artery. B-mode imaging was used to visualize graft structure and luminal opening. Color Doppler imaging was used to assess graft patency by confirming continuous blood flow through the graft segment. All measurements were performed under consistent probe positioning and acquisition settings to ensure reproducibility across time points.

**12.3 Histological and immunohistochemical analysis**

At 1, 3, 6, and 21 weeks post-implantation, rats were anesthetized and the implanted vascular grafts were re-exposed through a midline cervical incision. Grafts with adjacent native arterial tissue were carefully dissected and gently rinsed with heparinized saline (180 U mL⁻¹) to remove residual blood [14]. Samples were fixed in 4% paraformaldehyde (Electron Microscopy Sciences) at 4 °C for 2 h, followed by cryoprotection in 30% sucrose at 4 °C for 24 h. Explants were embedded in optimal cutting temperature (OCT) compound, snap-frozen at −80 °C, and cryosectioned into 8 μm-thick transverse sections. Sections were processed at the Animal Health Diagnostic Center (Cornell University) for histological staining, including hematoxylin and eosin (H&E), Masson’s trichrome, Verhoeff–Van Gieson elastin staining, and Alizarin Red staining. Immunohistochemical (IHC) staining was performed for calponin (smooth muscle cell marker) and von Willebrand factor (vWF, endothelial marker). Hematoxylin counterstaining was applied for 5 min to visualize nuclei.

Stained sections were scanned using a PreciPoint M8 digital slide scanner at 4×, 10×, and 20× objective magnifications. For quantitative analysis, H&E-stained cross-sections from four independent grafts per group (*n* = 4) at each time point were analyzed using ImageJ software (NIH). Luminal circumference was measured and converted to luminal radius (r = C / 2π), from which luminal area (A = πr²) was calculated. Graft wall thickness, defined as the combined intimal and medial layers, was determined by averaging measurements taken at six evenly distributed locations around the circumference of each section.

13 **Statistical Analysis**

All data were reported as the mean ± standard deviation (SD). One-way ANOVA statistical analysis was performed to evaluate the significance of the experimental data, followed by a Tukey’s post hoc test for pairwise comparison. All statistical analyses were executed using Kyplot 2.0 beta 15; a difference was considered significant when the p-value was less than 0.05 and the data were indicated with (*) for *p <* 0.05, (**) for *p <* 0.01 and (***) for *p <* 0.001, respectively.

References

[1] Yu SC, Hou S, Chan WK. Synthesis and properties of polyamides and polyesters on the basis of 2,2′-bipyridine-5,5′-dicarboxylic acid and the corresponding polymer-ruthenium complexes. Macromolecules. 2000, 33: 3259.

[2] Winkler M, Lacerda TM, Mack F, Meier MA. Renewable polymers from itaconic acid by polycondensation and ring-opening-metathesis polymerization. Macromolecules. 2015, 48: 1398.

[3] Guo W, Shen Z, Guo B, Zhang L, Jia D. Synthesis of bio-based copolyester and its reinforcement with zinc diacrylate for shape memory application. Polymer. 2014, 55: 4324.

[4] Chen Y, Miller PG, Ding X, Stowell CE, Kelly KM, Wang Y. Chelation crosslinking of biodegradable elastomers. Adv. Mater. 2020, 32: 2003761.

[5] Ji H, Yang H, Zhou X, Sun C, Li L, Zhao S, Yu J, Li S, Wang R, Zhang L. Preparation of bio-based elastomer and its nanocomposites based on dimethyl itaconate with versatile properties. Compos. Part B Eng. 2023, 248: 110383.

[6] Diehl B. NMR applications for polymer characterisation. In: NMR spectroscopy in pharmaceutical analysis. Amsterdam: Elsevier; 2008. pp. 157–180.

[7] Liu S-H, Guo R-L, Li F-H, Chiang C-L. Thermal analysis and pyrolysis products analysis of 4,4′-azobis(4-cyanovaleric acid) by using thermal analysis methods and combination technology: S.-H. Liu et al. J. Therm. Anal. Calorim. 2023, 148: 5009.

[8] Ding X, Gantumur N, Lee BP, Chen YG. Photocurable and 3D printable functional polyesters to engineer elastomeric scaffolds for biomedical applications. Macromol. Biosci. 2025: e00189.

[9] Cristiano MC, Froiio F, Mancuso A, De Gaetano F, Ventura CA, Fresta M, Paolino D. The Rheolaser Master™ and Kinexus rotational rheometer® to evaluate the influence of topical drug delivery systems on rheological features of topical poloxamer gel. Molecules. 2020, 25: 1979.

[10] Wang Y, Ameer GA, Sheppard BJ, Langer R. A tough biodegradable elastomer. Nat. Biotechnol. 2002, 20: 602.

[11] Kozbial A, Trouba C, Liu H, Li L. Characterization of the intrinsic water wettability of graphite using contact angle measurements: effect of defects on static and dynamic contact angles. Langmuir. 2017, 33: 959.

[12] Ding X, Chen YG, Goltz E, Gantumur N, Lee BP. Biodegradable synthetic graft with sustained hydrogen sulfide release promotes endothelial cell growth. Macromol. Biosci. 2025, 25: e00308.

[13] Chen WC, Wang Z, Missinato MA, Park DW, Long DW, Liu H-J, Zeng X, Yates NA, Kim K, Wang Y. Decellularized zebrafish cardiac extracellular matrix induces mammalian heart regeneration. Sci. Adv. 2016, 2: e1600844.

[14] Nguyen AK, Goering PL, Reipa V, Narayan RJ. Toxicity and photosensitizing assessment of gelatin methacryloyl-based hydrogels photoinitiated with lithium phenyl-2,4,6-trimethylbenzoylphosphinate in human primary renal proximal tubule epithelial cells. Biointerphases. 2019, 14.

[15] Chen YG, Dombaxe C, D’Amato AR, Van Herck S, Welch H, Fu Q, Zhang S, Wang Y. Transformation of metallo-elastomer grafts in a carotid artery interposition model over a year. Biomaterials. 2024, 309: 122598.

[16] Stowell CE, Li X, Matsunaga MH, Cockreham CB, Kelly KM, Cheetham J, Tzeng E, Wang Y. Resorbable vascular grafts show rapid cellularization and degradation in the ovine carotid. J. Tissue Eng. Regen. Med. 2020, 14: 1673.

[17] Tran RT, Naseri E, Kolasnikov A, Bai X, Yang J. A new generation of sodium chloride porogen for tissue engineering. Biotechnol. Appl. Biochem. 2011, 58: 335.

**Figure S1** Theoretical monomer feed compositions, design rationale, and code nomenclature for the PBIS polymer library. The table summarizes the molar feed percentages of 1,3-propanediol (PDO), diethyl [2,2′-bipyridine]-5,5′-dicarboxylate (Ethyl-DP), itaconic acid (IA), succinic acid (SU), and sebacic acid (SA) used for step-growth polymerization. Compositions were systematically varied to independently modulate backbone crystallinity (SU/SA ratio), metal-ligand coordination density (DP content), and covalent crosslinkable site density (IA content). The final column defines the code names used for each compositional series shown in Fig. 2. Percentages represent theoretical molar fractions in the monomer feed prior to polymerization.

| Batch | PDO% | Ethyl-DP% | IA% | SU% | SA% | Design variable | Code names |
| --- | --- | --- | --- | --- | --- | --- | --- |
| 1 | 100 | 10 | 10 | 0 | 80 | SA-only | PBIS__SA_ (Fig. 2a) |
| 2 | 100 | 10 | 10 | 40 | 40 | SU/SA=1/1;  medium DP;  medium IA | PBIS__SA/SU=1_ (Fig. 2a),  PBIS__DP-M_ (Fig. 2b),  PBIS__IA-M_ (Fig. 2c) |
| 3 | 100 | 10 | 10 | 80 | 0 | SU-only | PBIS__SU_ (Fig. 2a) |
| 4 | 100 | 5 | 10 | 42.5 | 42.5 | Low DP | PBIS__DP-L_ (Fig. 2b) |
| 5 | 100 | 20 | 10 | 35 | 35 | High DP | PBIS__DP-H_ (Fig. 2b) |
| 6 | 100 | 10 | 5 | 42.5 | 42.5 | Low IA | PBIS__IA-L_ (Fig. 2c) |
| 7 | 100 | 10 | 20 | 35 | 35 | High IA | PBIS__IA-H_ (Fig. 2c) |

**Figure S2** Representative quantitative ^1^H NMR spectrum of PBIS Batch 1 in CDCl_3_ showing peak assignments for the incorporated DP, IA, SA, and PDO units. Integrals were normalized to the PDO O-CH_2_ resonance (peak d, 4H per repeat unit). The bipyridine aromatic protons (h, i, j) and itaconate vinyl protons (g) were used to determine DP and IA contents, respectively, while aliphatic resonances (a–f) were assigned to sebacate, succinate, and PDO segments. The inset compares the theoretical composition with the actual composition obtained from ^1^H NMR analysis.

**Figure S3** Representative quantitative ^1^H NMR spectrum of PBIS Batch 2 in CDCl_3_ showing peak assignments for the incorporated DP, IA, SA, SU, and PDO units. Integrals were normalized to the PDO O-CH_2_ resonance (peak d, 4H per repeat unit). The bipyridine aromatic protons (h, i, j) and itaconate vinyl protons (g) were used to determine DP and IA contents, respectively, while aliphatic resonances (a–f) were assigned to sebacate, succinate, and PDO segments. The inset compares the theoretical composition with the actual composition obtained from ^1^H NMR analysis.

**Figure S4** Representative quantitative ^1^H NMR spectrum of PBIS Batch 3 in CDCl_3_ showing peak assignments for the incorporated DP, IA, SA, SU, and PDO units. Integrals were normalized to the PDO O-CH_2_ resonance (peak d, 4H per repeat unit). The bipyridine aromatic protons (h, i, j) and itaconate vinyl protons (g) were used to determine DP and IA contents, respectively, while aliphatic resonances (a–f) were assigned to sebacate, succinate, and PDO segments. The inset compares the theoretical composition with the actual composition obtained from ^1^H NMR analysis.

**Figure S5** Representative quantitative ^1^H NMR spectrum of PBIS Batch 4 in CDCl_3_ showing peak assignments for the incorporated DP, IA, SA, SU, and PDO units. Integrals were normalized to the PDO O-CH_2_ resonance (peak d, 4H per repeat unit). The bipyridine aromatic protons (h, i, j) and itaconate vinyl protons (g) were used to determine DP and IA contents, respectively, while aliphatic resonances (a–f) were assigned to sebacate, succinate, and PDO segments. The inset compares the theoretical composition with the actual composition obtained from ^1^H NMR analysis.

**Figure S6** Representative quantitative ^1^H NMR spectrum of PBIS Batch 5 in CDCl_3_ showing peak assignments for the incorporated DP, IA, SA, SU, and PDO units. Integrals were normalized to the PDO O-CH_2_ resonance (peak d, 4H per repeat unit). The bipyridine aromatic protons (h, i, j) and itaconate vinyl protons (g) were used to determine DP and IA contents, respectively, while aliphatic resonances (a–f) were assigned to sebacate, succinate, and PDO segments. The inset compares the theoretical composition with the actual composition obtained from ^1^H NMR analysis.

**Figure S7** Representative quantitative ^1^H NMR spectrum of PBIS Batch 6 in CDCl_3_ showing peak assignments for the incorporated DP, IA, SA, SU, and PDO units. Integrals were normalized to the PDO O-CH_2_ resonance (peak d, 4H per repeat unit). The bipyridine aromatic protons (h, i, j) and itaconate vinyl protons (g) were used to determine DP and IA contents, respectively, while aliphatic resonances (a–f) were assigned to sebacate, succinate, and PDO segments. The inset compares the theoretical composition with the actual composition obtained from ^1^H NMR analysis.

**Figure S8** Representative quantitative ^1^H NMR spectrum of PBIS Batch 7 in CDCl_3_ showing peak assignments for the incorporated DP, IA, SA, SU, and PDO units. Integrals were normalized to the PDO O-CH_2_ resonance (peak d, 4H per repeat unit). The bipyridine aromatic protons (h, i, j) and itaconate vinyl protons (g) were used to determine DP and IA contents, respectively, while aliphatic resonances (a–f) were assigned to sebacate, succinate, and PDO segments. The inset compares the theoretical composition with the actual composition obtained from ^1^H NMR analysis.

**Figure S9** Representative APC chromatograms of PBIS polymers synthesized across multiple batches. APC traces obtained using a refractive index (RI) detector are shown for Batch 1 and Batches 3–7. Each chromatogram exhibits a dominant primary polymer peak and, in several batches, a minor late-eluting secondary peak attributed to lower-molecular-weight oligomeric species. The reported weight-average molecular weight (*M_w_*) and polydispersity index (PDI) are presented as mean ± SD (*n* = 4) and were calculated from the principal polymer peak only, excluding the minor low-molecular-weight fraction.

**Figure S10** Representative DSC heating curves for Batch 1 and Batches 3–7, showing thermal transitions including glass-transition temperature (*T_g_*), crystallization temperature for Batch 1 (*T_c_*), cold-crystallization temperature for Batches 4–7 (*T_cc_*) where observed, and melting temperature (*T_m_*). The higher-temperature crystallization exotherm in Batch 1 is consistent with the enhanced crystallization tendency of the sebacic-acid-rich composition. Heat flow was normalized to sample mass (W/g).

**Figure S11** Representative TGA weight-loss curves of PBIS polymers synthesized across multiple batches. Curves for Batch 1 and Batches 3–7 show broadly consistent thermal degradation behavior. The temperature at 10% weight loss (*T_10%_*), maximum degradation temperature (*T_max_*), and residual mass at 495 °C are indicated for each batch.

**Figure S12** Quantitative tensile properties corresponding to the representative stress-strain curves shown in Fig. 2a. Bar plots summarize strain at fracture, ultimate tensile stress (UTS), Young’s modulus, and toughness for Fe-PBIS__SA_, Fe-PBIS__SA/SU = 1_, and Fe-PBIS__SU_ films. Data are presented as mean ± standard deviation (*n* = 4).

**Figure S13** Quantitative tensile properties corresponding to the representative stress-strain curves shown in Fig. 2b. Bar plots summarize strain at fracture, ultimate tensile stress (UTS), Young’s modulus, and toughness for Fe-PBIS__DP-L_, Fe-PBIS__DP-M_, and Fe-PBIS__DP-H_ films. Data are presented as mean ± standard deviation (*n =* 4).

**Figure S14** Quantitative tensile properties corresponding to the representative stress-strain curves shown in Fig. 2c. Bar plots summarize strain at fracture, ultimate tensile stress (UTS), Young’s modulus, and toughness for Fe-PBIS__IA-L_, Fe-PBIS__IA-M_, and Fe-PBIS__IA-H_ films. Data are presented as mean ± standard deviation (*n* = 4).

**Figure S15** Quantitative tensile properties corresponding to the representative stress-strain curves shown in Fig. 2d. Bar plots summarize strain at fracture, ultimate tensile stress (UTS), Young’s modulus, and toughness for PBIS__Fe+T_, PBIS__Fe+P_, PBIS__T_, PBIS__P_, and PBIS__Fe_. Data are presented as mean ± standard deviation (*n =* 4).

**Figure S16** Quantitative tensile properties corresponding to the representative stress-strain curves shown in Fig. 2e. Bar plots summarize strain at fracture, ultimate tensile stress (UTS), Young’s modulus, and toughness for Fe-PBIS, Cu-PBIS, Zn-PBIS, and Mg-PBIS elastomers. Data are presented as mean ± standard deviation (*n* = 4).

**Figure S17** Quantitative tensile properties corresponding to the representative stress-strain curves shown in Fig. 2k. Bar plots summarize strain at fracture, ultimate tensile stress (UTS), Young’s modulus, and toughness for Fe-PBIS films before and after self-healing. Data are presented as mean ± standard deviation (*n* = 4).

**Figure S18** Immunohistochemical evaluation of vascular graft remodeling at week 3 post-implantation. Representative cross-sectional images of contralateral common carotid artery (CCA), porous Zn-PBIS grafts (Zn-PBIS-P), and electrowritten Zn-PBIS grafts (Zn-PBIS-E). Staining includes von Willebrand factor (vWF) for endothelialization, Masson’s trichrome (MTS) for collagen deposition, α-smooth muscle actin (SMA) and calponin for smooth muscle cell phenotype, and Verhoeff elastin staining for elastic fiber formation (*n* = 4).

**Figure S19** Immunohistochemical evaluation of vascular graft remodeling at week 6 post-implantation. Representative cross-sectional images of contralateral common carotid artery (CCA), porous Zn-PBIS grafts (Zn-PBIS-P), and electrowritten Zn-PBIS grafts (Zn-PBIS-E). Staining includes von Willebrand factor (vWF) for endothelialization, Masson’s trichrome (MTS) for collagen deposition, α-smooth muscle actin (SMA) and calponin for smooth muscle cell phenotype, and Verhoeff elastin staining for elastic fiber formation (*n* = 4).
